# Identification of a Gene Prognostic Model of Gastric Cancer Based on Analysis of Tumor Mutation Burden

**DOI:** 10.3389/pore.2021.1609852

**Published:** 2021-09-10

**Authors:** Weijun Ma, Weidong Li, Lei Xu, Lu Liu, Yu Xia, Liping Yang, Mingxu Da

**Affiliations:** ^1^ School of Clinical Medicine, Ningxia Medical University, Yinchuan, China; ^2^ Department of Surgical Oncology, Gansu Provincial Hospital, Lanzhou, China; ^3^ The First Clinical Medical College, Gansu University of Chinese Medicine (Gansu Provincial Hospital), Lanzhou, China; ^4^ First Clinical Medical School, Lanzhou University, Lanzhou, China

**Keywords:** prognosis, gastric cancer, TCGA, immune infiltration, tumor mutation burden

## Abstract

**Introduction:** Gastric cancer is one of the most common cancers. Although some progress has been made in the treatment of gastric cancer with the improvement of surgical methods and the application of immunotherapy, the prognosis of gastric cancer patients is still unsatisfactory. In recent years, there has been increasing evidence that tumor mutational load (TMB) is strongly associated with survival outcomes and response to immunotherapy. Given the variable response of patients to immunotherapy, it is important to investigate clinical significance of TMB and explore appropriate biomarkers of prognosis in patients with gastric cancer (GC).

**Material and Methods:** All data of patients with gastric cancer were obtained from the database of The Cancer Genome Atlas (TCGA). Samples were divided into two groups based on median TMB. Differently expressed genes (DEGs) between the high- and low-TMB groups were identified and further analyzed. We identified TMB-related genes using Lasso, univariate and multivariate Cox regression analysis and validated the survival result of 11 hub genes using Kaplan-Meier Plotter. In addition, “CIBERSORT” package was utilized to estimate the immune infiltration.

**Results:** Single nucleotide polymorphism (SNP), C > T transition were the most common variant type and single nucleotide variant (SNV), respectively. Patients in the high-TMB group had better survival outcomes than those in the low-TMB group. Besides, eleven TMB-related DEGs were utilized to construct a prognostic model that could be an independent risk factor to predict the prognosis of patients with GC. What’s more, the infiltration levels of CD4^+^ memory-activated T cells, M0 and M1 macrophages were significantly increased in the high-TMB group compared with the low-TMB group.

**Conclusions:** Herein, we found that patients with high TMB had better survival outcomes in GC. In addition, higher TMB might promote immune infiltration, which could provide new ideas for immunotherapy.

## Introduction

Gastric cancer is the fifth most common cancer and the third leading cause of cancer-related deaths worldwide, resulting in more than one million new cases and nearly eighty thousand deaths in 2018 (1). Stomach adenocarcinoma accounts for the majority of all pathological types. Despite substantial advances in cancer treatment in recent decades, the overall survival rate of GC unfortunately remains unsatisfactory (1, 2). To date, the specific mechanisms that cause the onset and progression of GC remain unclear. Therefore, it is vital to investigate the underlying mechanism of GC progression as well as to find novel diagnostic and prognostic biomarkers.

Recently, immunotherapy has been regarded as a revolutionary treatment of malignant neoplasms (3, 4). For instance, personalized cancer vaccines, nanomaterials enhancing effect of T-cell, CAR-engineered T cells and immune checkpoint inhibitors (ICIs) have been greatly developed to overcome difficulties in treating cancer patients (5-7). Particularly, the emergence of ICIs has brought about a revolution in the treatment of multiple cancers. ICIs, including anti-PD1 inhibitors, pembrolizumab (8) and nivolumab (9) and the anti-CTLA4 inhibitor, ipilimumab (10), can considerably improve the survival outcomes in patients with cutaneous melanoma. However, the therapeutic efficacy of ICIs varies among cancer patients, and there is an urgent need to acquire effective biomarkers to guide the use of ICIs in cancer treatment. Fortunately, precision-targeted therapy has been widely recognized as a promising method against cancer due to ongoing discoveries of molecular mechanisms in tumorigenesis. The current view has described cancers as a kind of genomic diseases driven by an accumulation of both germline derived and somatic mutations. TMB is defined as the total of somatic mutations in every million bases (11). Present studies have shown a clear association between TMB and the outcome of immunotherapy for multiple cancer types (12, 13). On the one hand, mutations in driver genes are detrimental to drive tumorigenesis in humans, but on the other a large number of somatic mutations may generate many new antigens, which could be recognized and attacked by immune cells (14-16). In multiple cancers, high TMB had shown a close association with outcomes in patients using ICIs, such as non-small cell lung cancer (NSCLC) (17-19), breast cancer (20, 21), and melanoma (22).

TCGA database, which is benefitting from breakthroughs of high-throughput sequencing, contains plenty of precious bioinformatic sources including TMB available to the public. Bioinformatics analysis is a scientific method that has gained popularity in a wide range of fields, including oncology. Therefore, the present study aims to explore the significance of TMB in GC and construct a prognostic model by analyzing data from TCGA database.

## Materials and Methods

### Somatic Mutation Data Collection and Analysis

Masked Somatic Mutation datasets of 443 samples with GC were obtained from the TCGA database (https://portal.gdc.cancer.gov). The “maftools” R package was applied to perform further analysis and visualization of the data processed by the Mutect software. Transcriptome data (HTSeq-FPKM) and clinical data were also obtained from TCGA database. All these data are available to the public.

### Calculation of TMB and Analysis of Clinical Characteristics

TMB, the total of somatic mutations, including base substitutions, insertions or deletions in per million bases, was calculated by the total number of variants/the length of exons (the length of exons is 38 million bases) (13, 23). According to the median value of TMB, patients were divided into low- and high-TMB groups. The difference in survival between the two groups was evaluated by the Kaplan-Meier analysis using the log-rank test. In addition, differences in the TMB levels between clinical subgroups were tested by the Wilcoxon test or Kruskal-Wallis.

### Identification of Differential Expression Genes and Functional Analysis

We normalized the expression data using the “limma” package and identified DEGs between the low- and high-TMB groups by setting∣log2FC∣>1 and False Discovery Rate (FDR) < 0.05. The “pheatmap” R package was used to draw the volcano plot and heatmap of genes. Then, GO and KEGG pathway analysis of DEGs were performed with *p* < 0.05 and *Q* < 0.05 using “clusterProfiler,” “org.Hs.eg.db,” and “ggplot2” R packages.

### Calculation of Risk Score

We merged the survival time into the expression data of DEGs, and then screened 107 prognosis-related DEGs by univariate Cox with *p* < 0.05 as a screening condition, and further screened 11 of them as the most useful prognostic genes by Lasso Cox analysis using “glmnet” R package. For constructing the prognostic model, we obtained the respective coefficients of 11 prognosis-related DEGs based on multivariate Cox regression and calculated the risk score of each sample as follows: risk score = $\overset{11}{\underset{i=1}{\sum }}\left(exp_{i}\times coef_{i}\right)$. According to the median cutoff of risk score, patients were stratified into low- and high-risk groups, and the Kaplan-Meier analysis was conducted to compare the survival difference between two groups using “survival” and “survminer” packages. Finally, the prognostic significance of the prognostic model for GC was evaluated using the receiver operating characteristic curve (ROC), along with univariate and multivariate Cox regression analysis.

### Survival Validation of 11 Hub Genes

Kaplan-Meier Plotter (https://kmplot.com) was used to validate survival results of 11 genes. We selected all datasets for gastric cancer (GSE14210, GSE15459, GSE22377, GSE29272, GSE51105, GSE62254) and drew Kaplan-Meier plots with default parameters.

### Estimation of Immune Infiltration

The CIBERSORT algorithm was conducted to evaluate the immune fraction of each sample derived from TCGA by analyzing normalized gene expression data according to a known set providing a reference of transcriptome features of 22 types of immune cells (24). We then obtained an estimation of the abundances of member cell types in a mixed cell population. The different levels of immune infiltration of 22 types of immune cells between the low- and high-TMB group were tested by the Wilcoxon test, and the result was visualized by violin plots generated using “vioplot” R package. In addition, TIMER server was used to evaluate the correlations between the expression of 11 hub genes and the immune infiltration level of B cells, CD8^+^ T cells, CD4^+^ T cells, macrophages, neutrophils and dendritic cells in stomach adenocarcinoma.

### Statistical Analysis

R (version 4.0.3) was used for all analyses. The Cox regression model was constructed by the “survival” package, and the normalization and differential analysis were carried out by “Limma” package. Kaplan-Meier analysis was utilized to compare survival differences. Significance tests were performed using Wilcoxon’s test for two groups and Kruskal-Wallis’ test for three or more groups. Differences were statistically significant at *p* < 0.05.

## Results

### Analysis of the Mutation Features in GC

**FIGURE 1:**
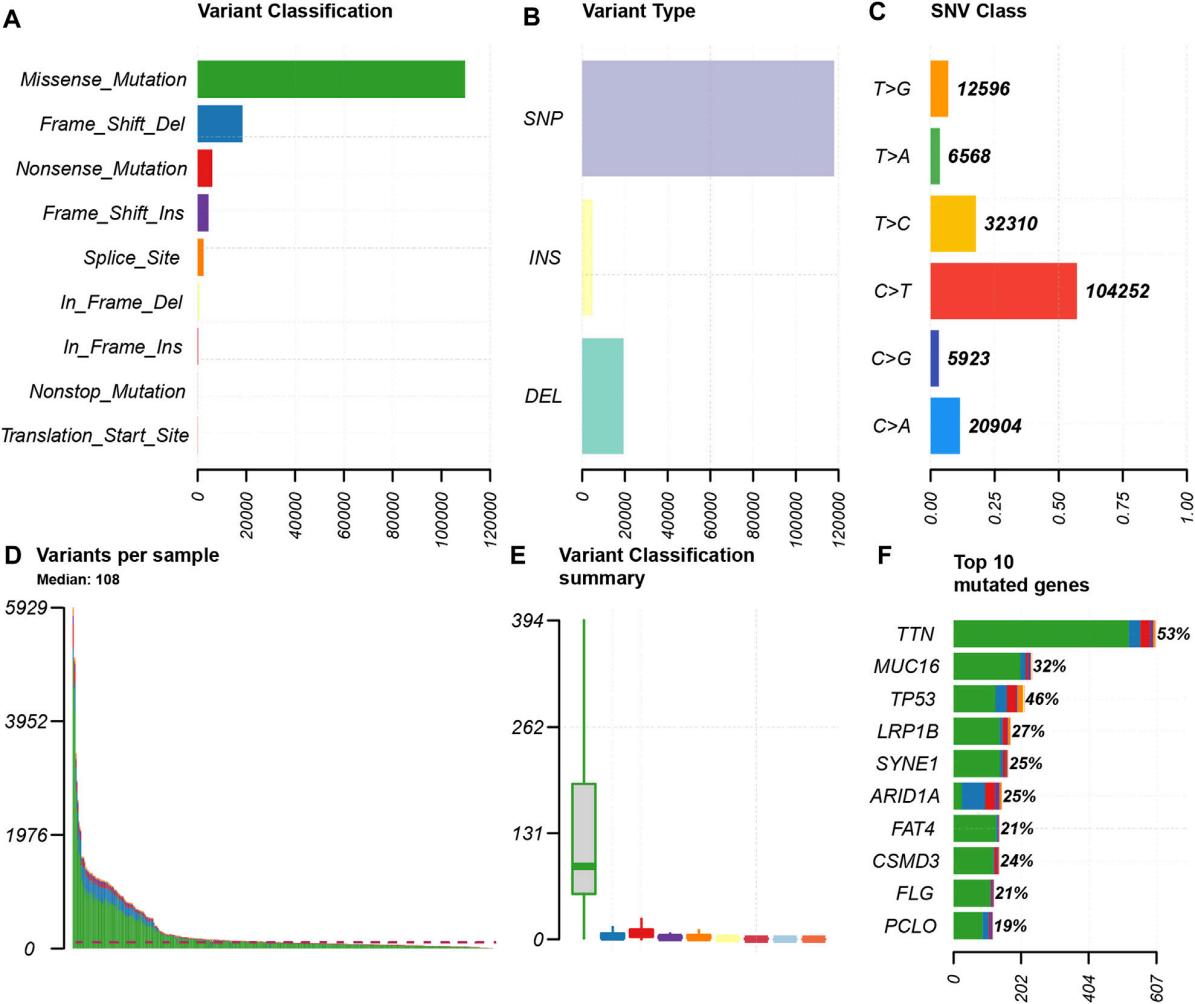
Summary of the mutation information. **(A)** Missense mutation was the most common of variant classification because of the highest frequency; **(B)** SNP occurred most frequently in variant types because of the highest frequency; and **(C)** C > T accounted for the most fraction in SNV; **(D and E)** the number of tumor mutation burden in specific samples; **(F)** the top 10 mutated genes in GC.

**FIGURE 2:**
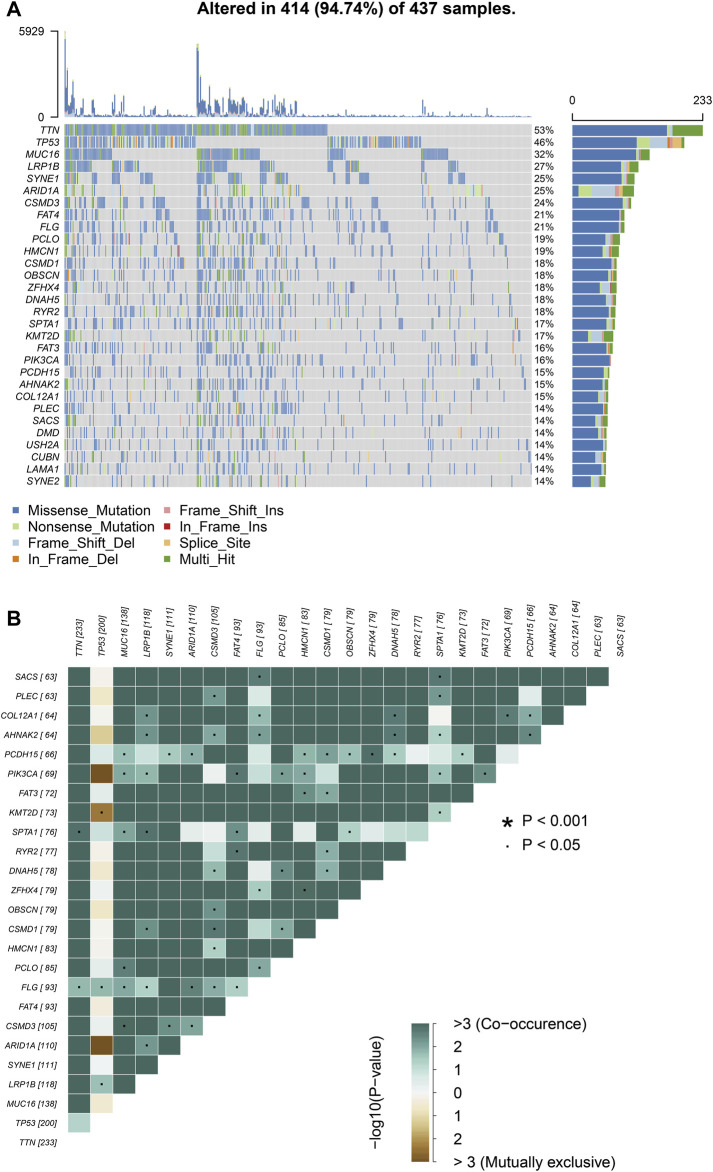
Landscape of mutation profiles in GC samples. **(A)** Mutation information of top 30 genes in each sample. The mutation frequency of each gene was shown via the bar plot at the right and the number of mutation burden was exhibited in the bar plot above the legend. **(B)** The coincident and exclusive relationship among mutated genes.

We choose mutation profiling processed by Mutect software and performed further analysis using the “maftools” package. The results showed that missense mutation, single nucleotide polymorphism, and C > T transition predominated in variant classification, variant type and SNV class, respectively in GC (Figures 1A–C). We got the number of altered bases from each patient, and differentiated mutation types with various colors in box plot (Figures 1D,E). According to the mutation frequency, we listed the top 10 mutated genes including *TTN* (53%), *MUC16* (32%), *TP53* (46%), *LRP1B* (27%), *SYNE1* (25%), *ARID1A* (25%), *FAT4* (21%), *CSMD3* (24%), *FLG* (21%), *PCLO* (19%) in GC (Figure 1F). Besides, the particular mutation information of top 30 mutated genes in each patient was visualized by a waterfall plot (Figure 2A), and Figure 2B showed the coincident and exclusive associations among these genes.

### Clinical Significance of TMB

**FIGURE 3:**
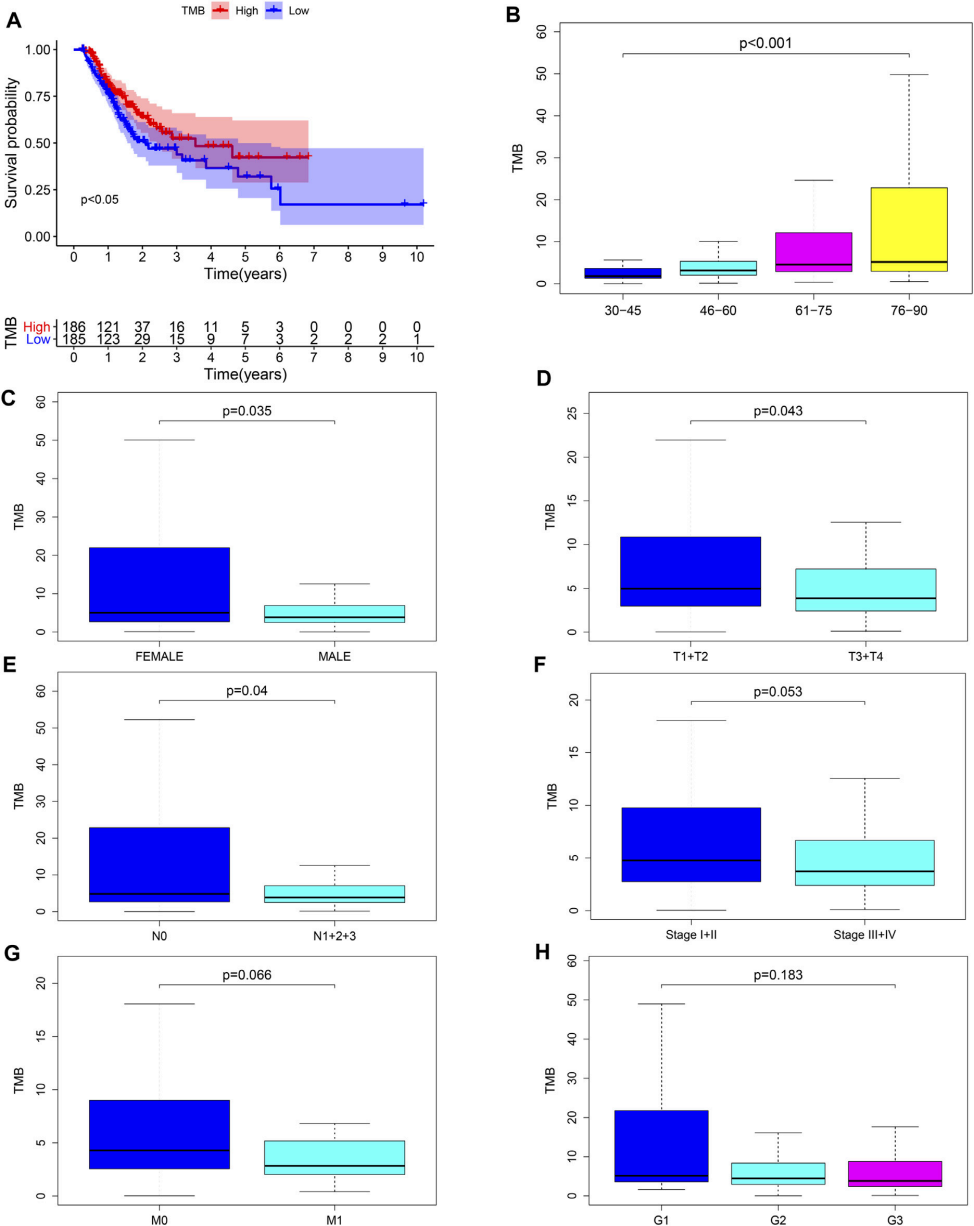
Associations between TMB and clinical characteristics. **(A–E)** TMB was significantly associated with survival rate (*p* < 0.05), age (*p* < 0.001), sex (*p* = 0.035), T (*p* = 0.043) and N stages (*p* = 0.04); **(F)** association between TMB and pathological stages was near to statistical significance; **(G–H)** no significant correlation between TMB and M stages or tumor grades.

We calculated the TMB value of each sample via Perl, and divided samples into low- and high-TMB groups based on the median of TMB. Then, we downloaded clinical datasets of 443 samples from TCGA, including survival status, survival time, age, gender, AJCC-TNM stage and pathological stage, and the baseline data were summarized in Table 1. After merging TMB values and clinical data, we evaluated the survival differences between low- and high-TMB groups by the Kaplan-Meier analysis. The results showed that patients with high TMB had a greater survival possibility compared with those with low TMB (Figure 3A). The TMB level was significantly higher in patients aged 60 years or older (*p* < 0.001) (Figure 3B), female (*p* = 0.035) (Figure 3C), and in earlier T and N stages (Figures 3D,E). Besides, the difference in the TMB level between pathological stages were slightly below the range of significance (*p* = 0.053) (Figure 3F). However, no significant differences in the TMB level were found in tumor grades or M stages (Figures 3G,H).

**TABLE 1:** Clinical baseline of 443 GC patients included in study from TCGA cohort.

Variables	Number (%)
Total	443 (100)
Status	
Alive	290 (65.46)
Dead	153 (34.54)
Age (known)	65.68 ± 10.76
Gender	
Female	158 (35.67)
Male	285 (64.33)
T	
T1+T2	116 (26.19)
T3+T4	317 (71.56)
Tx	10(2.25)
N	
N0	132 (29.80)
N1+2 + 3	292 (65.91)
Nx	19 (4.29)
M	
M0	391 (88.26)
M1	30 (6.77)
Mx	22 (4.97)
Tumor grade	
G1	12 (2.71)
G2	159 (35.89)
G3	263 (59.37)
Unknown	9 (2.03)
Stage	
Stage I and II	189 (42.66)
Stage III and IV	227 (51.24)
Unknown	27(6.10)

Tx, Nx, and Mx indicated that the situation could not be accurately assessed

### Differences of Gene Expression Between Low- and High-TMB Groups

**FIGURE 4:**
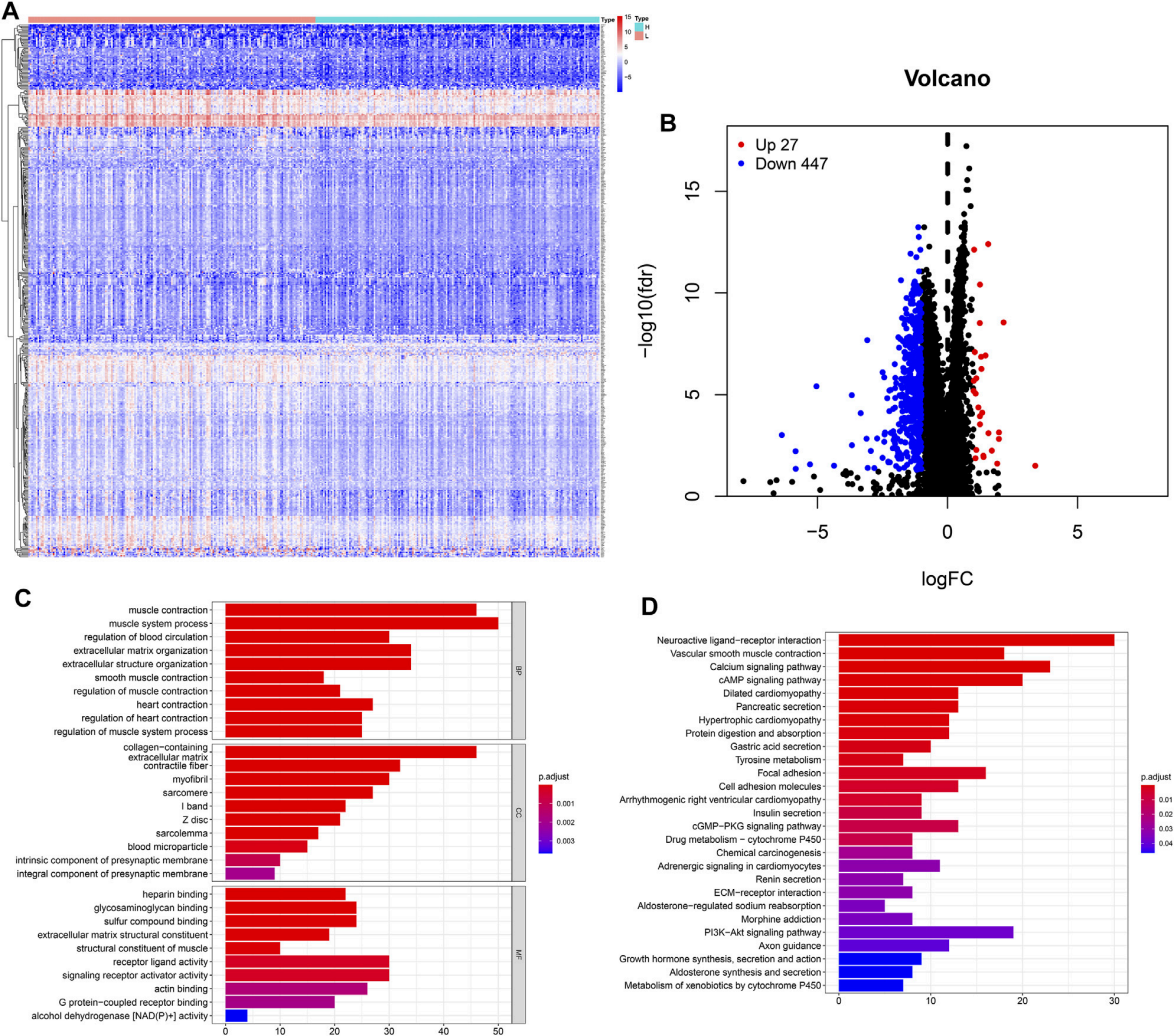
Features of the expression of genes in low- and high-TMB samples. **(A)** Differential expression of 474 TMB-related DEGs were exhibited by heatmap plot (the high-TMB group was indicated as “H,” while the low-TMB was indicated as “L”); **(B)** The volcano plot showed a total of 474 TMB-related DEGs, including 27 upregulated and 447 downregulated genes; **(C)** GO analysis showed top 10 results in biological process, cellular component and molecular function, respectively. **(D)** KEGG analysis indicated important pathways, in which DEGs significantly involved.

The heatmap showed that genes were commonly down regulated in the high-TMB group (Figure 4A). The volcano plot indicated that 474 DEGs were identified (Table S1), including 27 upregulated and 447 downregulated genes (Figure 4B). Furthermore, GO enrichment analysis revealed that DEGs were related to muscle system process, extracellular matrix, signaling receptor activator activity and so on (Figure 4C), while KEGG analysis showed that DEGs mainly participated in Neuroactive ligand-receptor interaction, Calcium signaling, cAMP signaling, cGMP-PKG signaling, PI3K-Akt signaling pathways (Figure 4D).

### Construction and Assessment of the Prognostic Model for Gastric Cancer

**FIGURE 5:**
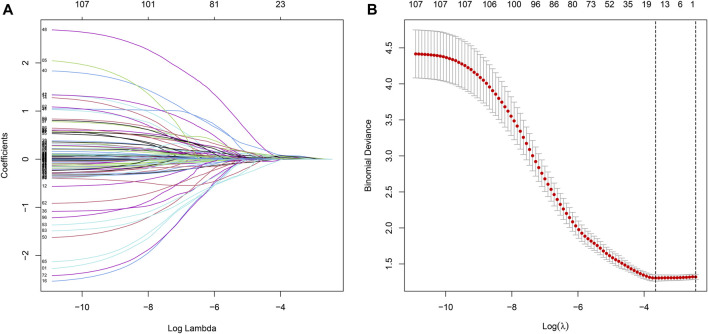
Prognostic DEGs related to TMB are identified by the Lasso Cox.

**FIGURE 6:**
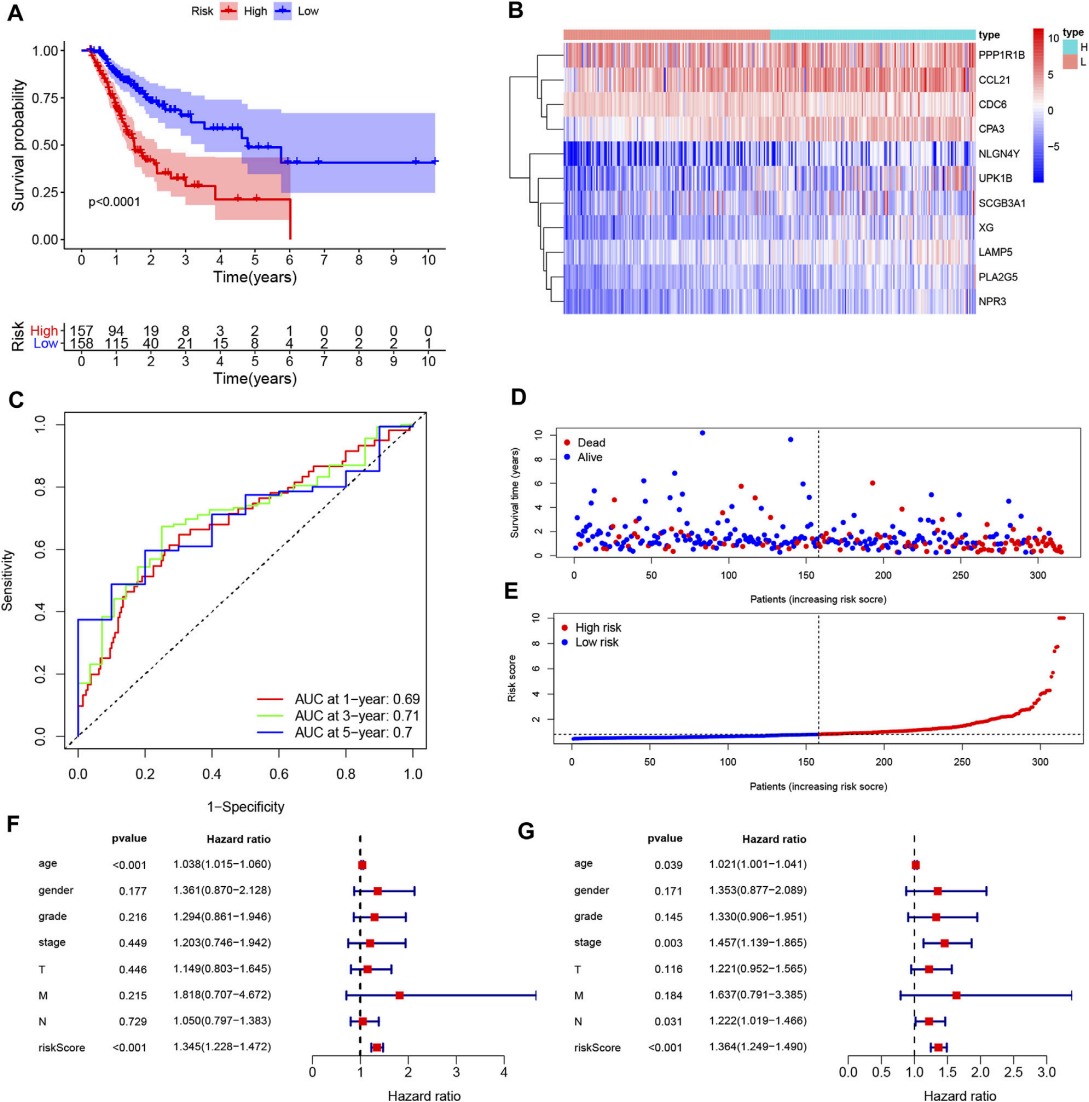
Construction and assessment of the prognosis model for GC. **(A)** Survival difference between low- and high-risk group was shown by the Kaplan-Meier curve. **(B)** The ROC curves of the prognostic model for 1, 3, and 5 years; **(C)** Heatmap of 11 genes for constructing the prognostic model; **(D,E)** the distribution of the risk score, and the survival status of patients; **(F,G)** Univariate Cox **(F)** and multivariate Cox **(G)** demonstrated that the risk score could be an independent prognostic factor for GC.

**FIGURE 7:**
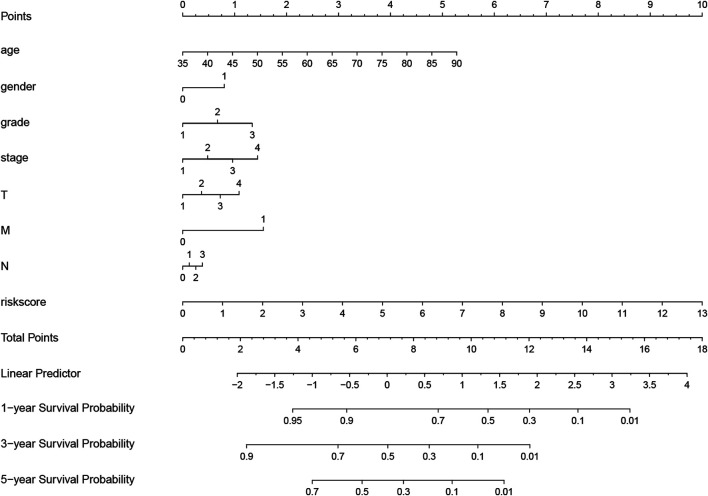
Nomogram for evaluating survival possibility at 1, 3, and 5-year for GC patients. “0” and “1” represented female and male respectively. Grade, stage and TNM were quantified based on corresponding clinical classification.

Univariate Cox regression was used to identify genes related to prognosis and 107 DEGs were selected for further analysis (Table S2). We further screened genes for construction of the prognostic model by Lasso Cox regression analysis (Figures 5A,B). Then, the multivariate Cox regression analysis were performed to calculate respective coefficients of these genes and the results were listed in Table 2. Based on these results, we calculated the risk score of each sample as follows: risk score = (*SCGB3A1* ×0.001394) + (*UPK1B* ×0.023706) + (*XG* ×0.122,584) + (*CCL21* ×0.000662) + (*CDC6* ×0.004405) + (*PLA2G5* ×0.189,744) + (*LAMP5* ×0.036008) + (*NLGN4Y* ×0.251,365) + (*NPR3* ×0.097112) + (*CPA3* ×0.012013) + (*PPP1R1B* ×0.000939). According to the median of all scores, low- and high-risk groups were established to differentiate patients, and patients with low-risk exhibited a better survival outcome than those with high-risk based on the Kaplan-Meier analysis (Figure 6A). Besides, we analyzed expression differences of 11 genes by heatmap (Figure 6B) and visualized the distribution of the risk and survival status for patients (Figures 6D,E). For evaluating the predictive accuracy of the prognostic model, the ROC curve was carried out with AUCs up to 0.69, 0.71, and 0.70 for 1, 3, and 5-year OS, respectively (Figure 6C), while univariate and multivariate Cox proportional hazard models demonstrated that the risk score was an independent prognostic factor for GC (Figures 6F,G). Last, based on clinical parameters and risk level, we calculated a score for each variable and predicted 1, 2, and 3-year survival probabilities by the total point of all variables. The nomogram indicated lower survival probabilities when the total point gradually accumulated (Figure 7).

**TABLE 2:** The result of multi-Cox regression of TMB-related signatures.

Gene	Coef	HR	HR.95L	HR.95H	*p* value
SCGB3A1*	0.001394	1.001395	1.000364	1.002427	0.007985
UPK1B*	0.023706	1.023989	1.010333	1.03783	0.000539
XG*	0.122,584	1.130,414	1.043159	1.224,968	0.002782
CCL21	0.000662	1.000662	0.999,954	1.001371	0.067021
CDC6*	0.004405	1.004414	1.001204	1.007635	0.007009
PLA2G5*	0.189,744	1.20894	1.10802	1.319,052	0.00002
LAMP5	0.036008	1.036665	0.993,804	1.081373	0.094627
NLGN4Y	0.251,365	1.285,779	0.953,986	1.73297	0.098814
NPR3*	0.097112	1.101,984	1.026028	1.183,564	0.007696
CPA3	0.012013	1.012086	0.998,593	1.025761	0.079379
PPP1R1B*	0.000939	1.00094	1.000293	1.001587	0.004378

### Survival Validation of 11 Hub Genes in GEO Datasets

**FIGURE 8:**
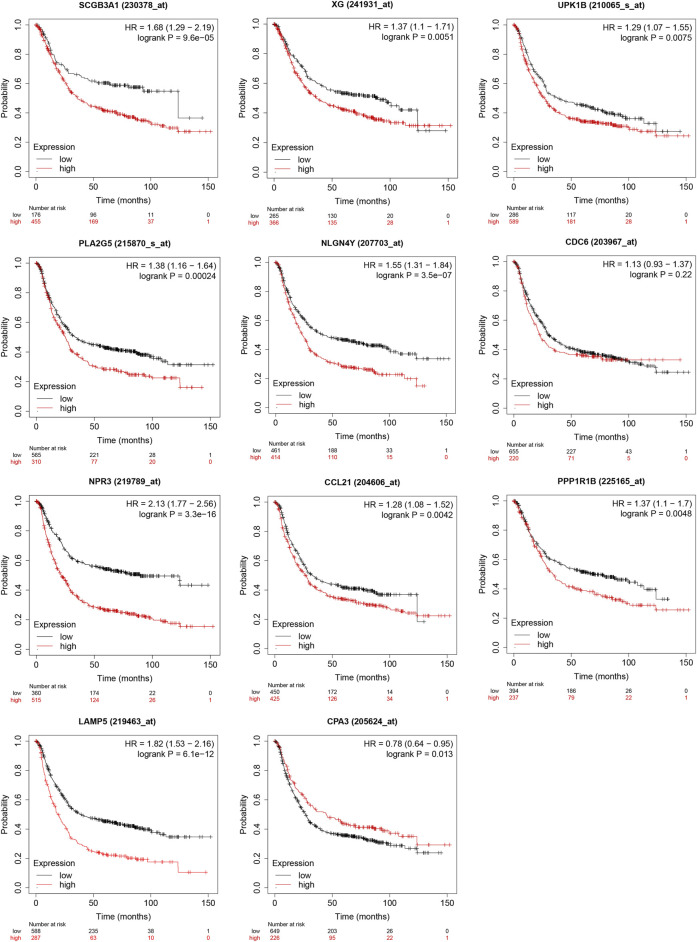
Validation of survival results of 11 prognostic signatures in GEO Datasets by Kaplan-Meier Plotter.

The result from Kaplan-Meier Plotter showed a better OS in the low-expression group of *SCGB3A1*, *UPK1B*, *XG*, *CCL21*, *PLA2G*, *LAMP5*(*C20orf103*), *NLGN4Y*, *NPR3*, *PPP1R1B* and the high-expression group of *CPA3*, which could be prognostic biomarkers (Figure 8). However, there was no significant difference in overall survival between two groups of *CDC6* when all datasets were selected.

### Differences of Immune Infiltration Level Between High- and Low-TMB Groups

**FIGURE 9:**
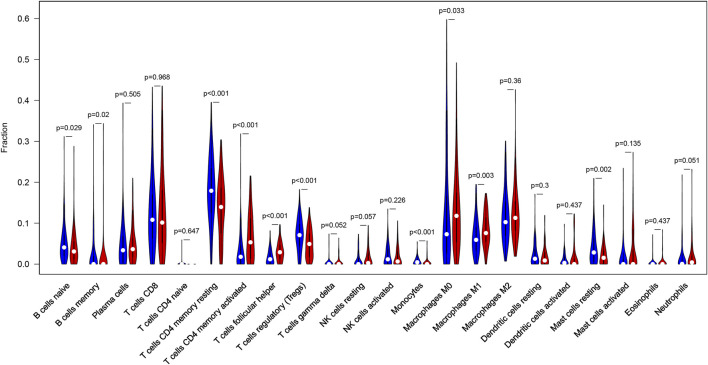
Differential infiltration levels of 22 types of immune cells in low- and high-TMB groups. High-TMB group and Low-TMB group are indicated as red and blue, respectively.

CIBERSORT algorithm was constructed to obtain the infiltration fractions of 22 types of immune cells. After selecting results at *p* < 0.05, the Wilcoxon test was used to compare differences between the two groups. The final result was visualized by violin plots (Figure 9), indicating that the infiltration levels of memory B cells, activated CD4^+^ memory T cells, follicular helper T cells, M0 and M1 macrophages were higher in the high-TMB group than in the low-TMB group, while naive B cells, resting CD4^+^ memory T cells, regulatory T cells (Tregs), monocytes and resting mast cells showed opposite results.

### Correlations Between the Expression of 11 Hub Genes and Immune Infiltration

**FIGURE 10:**
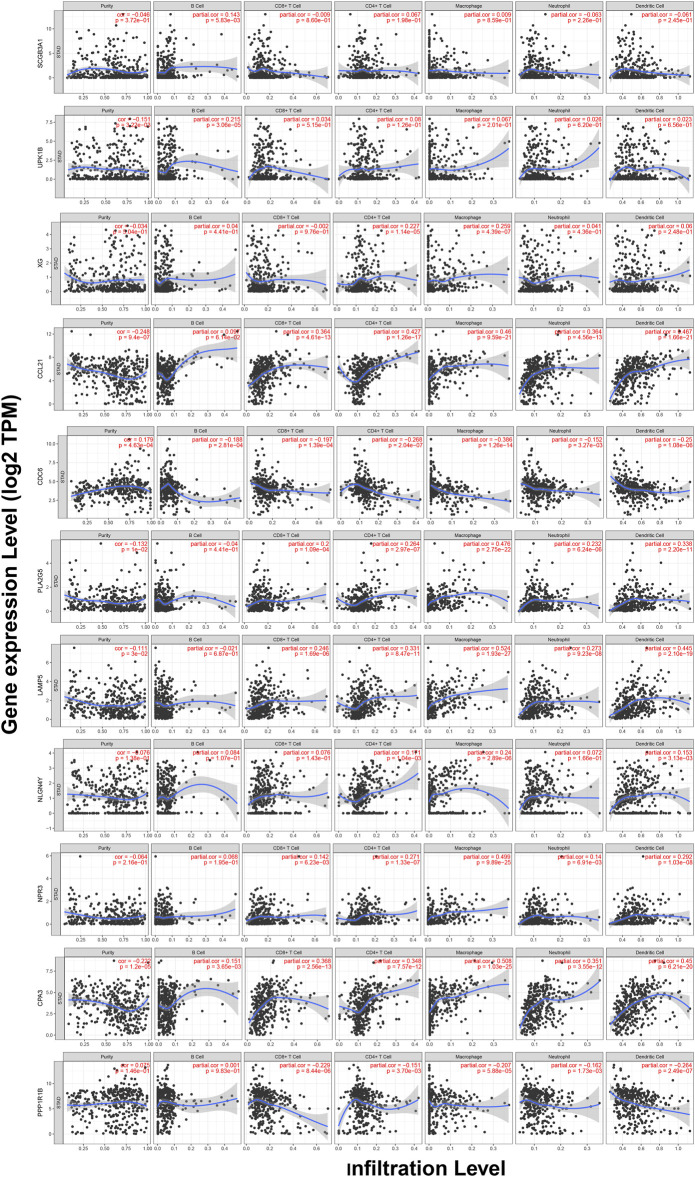
Correlations between the expression of 11 hub genes and immune infiltration in GC.

TIMER web server was used to analyze the function of hub genes in the immune infiltration. As shown in Figure 10 and Table S3, we found that the expression of *CCL21*, *PLA2G5*, *LAMP5*, *NPR3*, and *CPA3* was positively correlated with the infiltration level of CD8^+^ T cells, CD4^+^ T cells, macrophages, neutrophils and dendritic cells (including *SCGB3A1*, *UPK1B* and *CPA3* with B cells, *XG* with CD4^+^ T cells and macrophages, *NLGN4Y* with CD4^+^ T cells, macrophages and dendritic cells) (*p* < 0.05). In addition, the expression of *CDC6* and *PPP1R1B* was negatively correlated with the infiltration level of CD8^+^ T cells, CD4^+^ T cells, macrophages, neutrophils and dendritic cells (including *CDC6* with B cells) (*p* < 0.05) (Figure 10, Table S3).

## Discussion

With development of molecular biology in immunotherapy, many promising results have been achieved in the treatment of advanced cancers recently. Immune checkpoint inhibitors are applied in the treatment for chemo-refractory gastric cancer. A randomized trial showed antiPD1 monoclonal antibody nivolumab improved overall survival of patients with GC (25). Unfortunately, not all patients will respond well to such an immunotherapy. Therefore, it is of great clinical value to identify biomarkers that help to distinguish patients who could benefit from immunotherapy. TMB is a potential biomarker for predicting the outcome of immunotherapy in a variety of cancers (12, 26, 27). Somatic mutations are important causes of tumorigenesis, which lead to neoantigens that are recognized and attacked by immune cells (16). *TTN*, encoding a structural protein in striated muscles, is frequently detected in many tumors, which is associated with an increase in TMB and the objective response to ICIs (28, 29). *MUC16*, which encodes the cancer antigen CA-125, was shown to be strongly associated with higher TMB and favorable survival outcomes in GC patients (30). Our result illustrated that GC patients with high TMB had a higher survival rate than those with low TMB, which was consistent with the result of Zhao et al. (31), Yu et al. (32) and Wang et al. (33). Besides, higher TMB was associated with late T and N stages. Similarly, patients with high TMB had significantly higher response rates to ICIs and longer survival than those with low TMB (17). In NSCLC, it had been demonstrated that patients with high plasma TMB got significant benefits from the treatment of atezolizumab, particularly the benefit in PFS (34, 35). In addition, we identified 11 TMB-related genes including *SCGB3A1*, *UPK1B, XG, CCL21, CDC6, PLA2G5, LAMP5, NLGN4Y, NPR3, CPA3, PPP1R1B* and constructed a prognostic model showing poor prognosis in patients with high-risk score. Notably, the AUCs of ROC analysis for the prognostic model at 1, 3, and 5-year were 0.69, 0.71, and 0.70, respectively. SCGB3A1 (HIN1), secretoglobin 3A1 (a small secreted protein), is a member of the secretoglobin family (36). A previous report suggested that *SCGB3A1* expression was positively correlated with the level of B-cell infiltration (37), which was consistent with the results of the present study. Methylation of its promoter had been reported to be associated with poor outcome in patients with ovarian clear cell adenocarcinoma, and expression of the *SCGB3A1* gene can increase paclitaxel sensitivity via the Akt pathway (38). UPK1B, a member of the transmembrane four superfamily, was significantly associated with the prognosis and promoted the proliferation, migration and invasion by the Wnt/β-catenin signaling pathway in bladder cancer (39). *XG*, encoding the XG blood group antigen, was associated with lower OS in patients with Ewing’s sarcoma and played a role in metastasis (40). CCL21 is a chemokine that can affect lymph node metastasis in various cancer types. The expression of CCL21/CCR7 was significantly associated with colorectal liver metastasis (41). CDC6, cell division cycle 6, whose expression was promoted by zinc finger protein 143 (ZNF143) and accelerated hepatocellular carcinoma cell-cycle progression (42). PLA2G5 (phospholipase A2 group V) was reported to be associated with epithelial-mesenchymal transition and the isocitrate dehydrogenase one mutation status in gliomas (43). LAMP5, a lysosomal associated membrane protein, could directly target the oncogenic MLL-fusion protein, whose depletion lead to inhibition in leukemia cell growth *in vivo* and *in vitro* (44). NLGN4Y, a type I membrane protein that belonging to the family of neuroligins, was highly expressed in lung adenocarcinoma patients with poor survival (45). NPR3 (natriuretic peptide receptor 3), could inhibit cancer cells growth in osteosarcoma via blocking the PI3K/AKT pathway (46). CPA3 (carboxypeptidase A3), was demonstrated to be involved in the histone hyperacetylation signaling pathway activated during differentiation of prostate epithelial cancer cells (47). *PPP1R1B* encodes protein phosphatase one regulatory inhibitor subunit 1B and the knockdown of *PPP1R1B* impaired the ability of lung metastases in pancreatic cancer cells in mice (48).

CIBERSORT, an emerging approach that integrates the deconvolution algorithm and genomic profiles, can calculate relative proportions of tumor-infiltrating immune cells, which performs better than immunohistochemistry-based analysis (24), and has been increasingly used to estimate the infiltration of immune cells due to its favourable performance (49-52). We applied CIBERSORT to access the infiltration level of 22 types of immune cells and found higher immune infiltration levels of memory B cells, CD4^+^ memory activated T cells, M0 and M1 macrophages in the high-TMB group. Li et al. demonstrated that CD4^+^ T cells could help activate M1 macrophages, and the infiltrate levels of CD4^+^ and CD8^+^ T cells were negatively correlated with tumor size in gastric cancer (53). Macrophages are antigen-presenting cells that have the ability to bind specifically to tumor cells and can directly phagocytose and deliver drugs to tumors (54). Many studies have implied that macrophages can exert anti-cancer effects through targeted drug delivery (55, 56). M1 macrophages can inhibit tumor growth, while M2 macrophages can promote tumor growth, so the enhancement of M1 macrophages polarization and inhibition of M2 macrophages polarization are regarded as an effective way to the treatment of tumors. Consistently, our study indicated that the immune infiltration level of M1 macrophages was increased in the high-TMB group, which showed a favorable prognosis. Meanwhile, we found lower infiltration levels of naive B cells, resting CD4^+^ memory T cells, regulatory T cells (Tregs), monocytes and resting mast cells in the high-TMB group. Gu Y et al. reported that the infiltration of B cells could increase lymph node metastases by the production of pathogenic IgG in breast cancer (57). Olkhanud et al. found that breast cancer metastasis was promoted by tumor-evoked regulatory B cells through converting resting CD4^+^ T cells to regulatory T cells (58). Mast cells may play a role in tumor progression by supporting angiogenesis (59). In melanoma, mast cell-derived hypoxia-inducible factor 1 (HIF-1) could exacerbate the growth of tumor. TP53 plays a critical role in the prevention of oncogenesis in several cancer types and has been shown to be involved in the physiological disruption of the M2 macrophages polarization process through the TP53/MDM2/c-MYC axis (60). Another research suggested that the inhibition of MDMX phosphorylation could prevent the reduced expression of P53, which hindered M2 polarization and promoted M1 polarization *in vivo*. Combining with our findings, the mutation of TP53 might be an important factor contributing to differential immune infiltration of macrophages. In general, we concluded the landscape of TMB in GC, analyzed the differences of TMB in different clinical subgroups and constructed a prediction model. Taken together, our study indicated TMB was closely associated with the prognosis of patients with GC. A novel prognostic model and nomogram were built to predict the prognosis of GC.

## Data Availability

Data can be acquired from the TCGA database (https://portal.gdc.cancer.gov). R and Perl codes (https://figshare.com/articles/software/R_and_Perl_script/15033447).
