# Supplementary material for: Identification of a Gene Prognostic Model of Gastric Cancer Based on Analysis of Tumor Mutation Burden
Source: Pathol Oncol Res. 2021 Sep 10;27:1609852. doi: 10.3389/pore.2021.1609852 (PMC8460769; doi:10.3389/pore.2021.1609852)
Supplement: Supplementary file 1 [file table1.docx]

| Table S1 474 differential expression genes between low- and high-TMB groups | | | | | |
| --- | --- | --- | --- | --- | --- |
| gene | conMean | treatMean | logFC | pValue | fdr |
| FILIP1 | 2.3507351 | 1.0080326 | -1.2215697 | 0.0000000 | 0.0000001 |
| RASGRP2 | 1.8831250 | 0.7749251 | -1.2810000 | 0.0000000 | 0.0000000 |
| PDE3A | 3.8744581 | 1.8863838 | -1.0383713 | 0.0000055 | 0.0000285 |
| GC | 16.1847362 | 4.6798685 | -1.7900940 | 0.0000005 | 0.0000035 |
| ADGRD1 | 0.9635362 | 0.4782060 | -1.0107067 | 0.0000001 | 0.0000006 |
| VTN | 21.2305649 | 6.0307033 | -1.8157446 | 0.0000000 | 0.0000000 |
| MYOC | 2.3827277 | 0.6561112 | -1.8606018 | 0.0030450 | 0.0069273 |
| TPSAB1 | 18.6735394 | 9.0520803 | -1.0446741 | 0.0000000 | 0.0000000 |
| ZSCAN18 | 1.7958614 | 0.8352266 | -1.1044365 | 0.0000000 | 0.0000000 |
| FCER2 | 1.7135501 | 0.7389733 | -1.2133943 | 0.0002540 | 0.0007972 |
| RERG | 3.5397931 | 1.4182958 | -1.3195066 | 0.0000000 | 0.0000000 |
| LIPF | 512.2678108 | 66.4161798 | -2.9472916 | 0.0025172 | 0.0058526 |
| KCNJ15 | 1.4544744 | 0.6870326 | -1.0820474 | 0.0006184 | 0.0017314 |
| SCGB3A1 | 16.9293132 | 5.5179129 | -1.6173289 | 0.0000071 | 0.0000360 |
| CAND2 | 1.2697013 | 0.6080543 | -1.0622169 | 0.0000000 | 0.0000000 |
| VIP | 4.2118723 | 0.7806266 | -2.4317573 | 0.0007637 | 0.0020785 |
| TMEM252 | 1.1382836 | 0.3661768 | -1.6362476 | 0.0000006 | 0.0000042 |
| SOX21 | 4.0796632 | 1.8632865 | -1.1306005 | 0.0000003 | 0.0000024 |
| FAM107A | 2.1980637 | 0.8643143 | -1.3466052 | 0.0000000 | 0.0000000 |
| DES | 572.0275605 | 139.7185857 | -2.0335607 | 0.0004413 | 0.0012936 |
| DNAJB5 | 4.1762664 | 1.7856932 | -1.2257295 | 0.0000005 | 0.0000037 |
| TMTC1 | 2.5607737 | 1.1254189 | -1.1861176 | 0.0000000 | 0.0000000 |
| SELP | 4.3843145 | 2.0977983 | -1.0634753 | 0.0000000 | 0.0000001 |
| ADH1B | 5.5387563 | 2.1035234 | -1.3967542 | 0.0000001 | 0.0000006 |
| DTNA | 0.9589564 | 0.4552901 | -1.0746793 | 0.0000001 | 0.0000007 |
| LMOD1 | 35.3074553 | 10.5430173 | -1.7436850 | 0.0000007 | 0.0000049 |
| SLCO1B3 | 0.4082478 | 1.0659179 | 1.3845794 | 0.0055575 | 0.0117049 |
| SORBS1 | 20.7632432 | 7.3879557 | -1.4907847 | 0.0000001 | 0.0000013 |
| RIPOR2 | 2.0356313 | 0.9817545 | -1.0520420 | 0.0000032 | 0.0000179 |
| ITGA8 | 4.0469195 | 1.5040217 | -1.4279987 | 0.0000000 | 0.0000000 |
| ASB2 | 4.0436856 | 1.4056983 | -1.5243839 | 0.0000001 | 0.0000006 |
| GLYATL2 | 1.6199402 | 0.1264474 | -3.6793317 | 0.0000017 | 0.0000106 |
| CSRP1 | 50.1833813 | 23.8359611 | -1.0740699 | 0.0000739 | 0.0002727 |
| BLK | 1.4317984 | 0.5449159 | -1.3937228 | 0.0000153 | 0.0000696 |
| NCAM1 | 1.3090304 | 0.4199655 | -1.6401557 | 0.0000002 | 0.0000016 |
| TCEAL7 | 2.0605061 | 0.9637735 | -1.0962326 | 0.0000000 | 0.0000001 |
| PDZRN3 | 6.4517621 | 3.0946781 | -1.0599039 | 0.0000000 | 0.0000001 |
| TAC1 | 0.9865550 | 0.1747586 | -2.4970357 | 0.0061522 | 0.0127893 |
| CHRDL2 | 19.3313600 | 9.1097720 | -1.0854563 | 0.0000016 | 0.0000100 |
| ZBTB16 | 1.2165486 | 0.3619516 | -1.7489254 | 0.0000000 | 0.0000000 |
| HP | 1.1350655 | 0.2277814 | -2.3170536 | 0.0000026 | 0.0000149 |
| FAM189A2 | 2.8455874 | 1.3990647 | -1.0242639 | 0.0000001 | 0.0000008 |
| PODN | 17.3330242 | 7.7506653 | -1.1611313 | 0.0000000 | 0.0000001 |
| GHRL | 5.0283939 | 0.9310735 | -2.4331307 | 0.0000002 | 0.0000014 |
| TNXB | 4.9724585 | 1.7804986 | -1.4816780 | 0.0000000 | 0.0000003 |
| HSPB7 | 13.5897406 | 3.9773260 | -1.7726472 | 0.0000071 | 0.0000356 |
| INHA | 1.6555787 | 0.6927084 | -1.2570155 | 0.0084583 | 0.0167867 |
| MPDZ | 2.0873384 | 1.0267611 | -1.0235639 | 0.0000000 | 0.0000000 |
| KCNMA1 | 4.2695846 | 1.3537498 | -1.6571346 | 0.0000023 | 0.0000134 |
| MYBPC1 | 2.8715126 | 1.2796822 | -1.1660253 | 0.0013080 | 0.0033201 |
| KIF1A | 1.9652109 | 0.7269434 | -1.4347691 | 0.0000080 | 0.0000400 |
| TNFSF9 | 3.2552692 | 7.7296721 | 1.2476304 | 0.0000782 | 0.0002869 |
| PRIMA1 | 3.3397011 | 0.9189277 | -1.8616958 | 0.0000000 | 0.0000000 |
| SMTN | 21.9829435 | 10.8659370 | -1.0165720 | 0.0119360 | 0.0225840 |
| SLIT3 | 4.3191238 | 1.9789712 | -1.1259881 | 0.0000000 | 0.0000001 |
| CAVIN2 | 9.9503967 | 3.7160256 | -1.4209936 | 0.0000000 | 0.0000000 |
| MAP1B | 8.8571047 | 3.8636398 | -1.1968746 | 0.0000000 | 0.0000000 |
| PYGM | 1.1040044 | 0.3500186 | -1.6572424 | 0.0000000 | 0.0000001 |
| NNAT | 5.5613106 | 1.6167632 | -1.7823165 | 0.0069108 | 0.0141456 |
| CHRM2 | 1.2737009 | 0.3343622 | -1.9295427 | 0.0005006 | 0.0014447 |
| ADAM23 | 1.0297971 | 0.4994603 | -1.0439183 | 0.0000000 | 0.0000000 |
| MYL9 | 275.9124941 | 97.5307837 | -1.5002812 | 0.0000323 | 0.0001322 |
| SST | 55.0191815 | 5.4436277 | -3.3372944 | 0.0000183 | 0.0000814 |
| RBP1 | 7.5700507 | 3.5553116 | -1.0903269 | 0.0000000 | 0.0000001 |
| TLR10 | 1.5785644 | 0.6206041 | -1.3468681 | 0.0001142 | 0.0003982 |
| KRTAP3-1 | 0.4817372 | 4.9889953 | 3.3724310 | 0.0175540 | 0.0314913 |
| NPY | 1.0942204 | 0.5349878 | -1.0323253 | 0.0022135 | 0.0052253 |
| SCTR | 1.3857191 | 0.6273180 | -1.1433661 | 0.0000000 | 0.0000001 |
| C16orf89 | 3.5250250 | 1.1341385 | -1.6360367 | 0.0000000 | 0.0000002 |
| FGG | 3.5146012 | 1.1950977 | -1.5562324 | 0.0002609 | 0.0008160 |
| NGFR | 3.9854203 | 1.7248776 | -1.2082379 | 0.0000000 | 0.0000001 |
| SCG5 | 6.5362558 | 1.6117111 | -2.0198713 | 0.0000088 | 0.0000433 |
| C8orf88 | 2.4075774 | 0.8984523 | -1.4220684 | 0.0000000 | 0.0000001 |
| GPRASP1 | 1.5611427 | 0.6361822 | -1.2950905 | 0.0000000 | 0.0000000 |
| MYLK | 25.0020398 | 8.2585839 | -1.5980795 | 0.0000019 | 0.0000115 |
| PLN | 16.1672896 | 5.7237077 | -1.4980559 | 0.0000365 | 0.0001470 |
| HSPB6 | 131.9543711 | 32.8427883 | -2.0063906 | 0.0000002 | 0.0000015 |
| GSTM5 | 0.7623951 | 0.3275815 | -1.2186849 | 0.0000000 | 0.0000000 |
| RBM24 | 0.7223199 | 0.3407057 | -1.0841118 | 0.0000011 | 0.0000073 |
| SPON1 | 22.1528551 | 10.8981767 | -1.0234059 | 0.0000122 | 0.0000569 |
| RERGL | 1.1082233 | 0.4943313 | -1.1646984 | 0.0000003 | 0.0000026 |
| COL14A1 | 14.5408571 | 5.6337967 | -1.3679329 | 0.0000000 | 0.0000000 |
| CH25H | 3.7220497 | 1.7961047 | -1.0512259 | 0.0000000 | 0.0000000 |
| GPR15 | 0.9640230 | 0.4432962 | -1.1207966 | 0.0000002 | 0.0000018 |
| ADAM33 | 2.3304165 | 0.8447851 | -1.4639315 | 0.0000000 | 0.0000001 |
| TPM2 | 94.3258758 | 36.8547020 | -1.3558049 | 0.0005440 | 0.0015503 |
| POPDC2 | 3.4240768 | 1.3017783 | -1.3952313 | 0.0000484 | 0.0001880 |
| ROR2 | 4.1203707 | 1.9763197 | -1.0599578 | 0.0000004 | 0.0000028 |
| CRP | 1.7859814 | 0.0868406 | -4.3622030 | 0.0174058 | 0.0312653 |
| LDB3 | 2.1203100 | 0.5905610 | -1.8441173 | 0.0000018 | 0.0000107 |
| ITIH2 | 2.7489802 | 1.0363212 | -1.4074252 | 0.0003927 | 0.0011683 |
| MYH11 | 175.6365395 | 43.6175791 | -2.0096114 | 0.0000025 | 0.0000145 |
| TAGLN | 159.7791723 | 60.2649773 | -1.4066876 | 0.0001183 | 0.0004106 |
| ITGA7 | 6.8312885 | 3.1912239 | -1.0980479 | 0.0000007 | 0.0000049 |
| FST | 4.9511921 | 1.9304011 | -1.3588753 | 0.0000052 | 0.0000275 |
| RGMA | 5.7490683 | 1.7576582 | -1.7096736 | 0.0000000 | 0.0000000 |
| BMP3 | 1.3661295 | 0.3734215 | -1.8712174 | 0.0000778 | 0.0002856 |
| SLC38A3 | 1.9264108 | 0.4494693 | -2.0996208 | 0.0002414 | 0.0007636 |
| NRXN3 | 1.3236786 | 0.4822367 | -1.4567394 | 0.0000524 | 0.0002017 |
| NACAD | 1.0662409 | 0.5140724 | -1.0524899 | 0.0000000 | 0.0000000 |
| LOXL4 | 2.1150665 | 1.0399410 | -1.0242014 | 0.0000000 | 0.0000000 |
| CILP | 3.9127192 | 1.9236617 | -1.0243165 | 0.0008220 | 0.0022155 |
| LTF | 36.3421210 | 13.2642767 | -1.4540966 | 0.0000080 | 0.0000397 |
| HSPB8 | 17.7592684 | 6.0232965 | -1.5599470 | 0.0000000 | 0.0000000 |
| ADH4 | 5.3524084 | 2.1971772 | -1.2845369 | 0.0057544 | 0.0120774 |
| UPK1B | 5.3529584 | 2.3940595 | -1.1608774 | 0.0000628 | 0.0002359 |
| IGF1 | 0.7976813 | 0.3410898 | -1.2256610 | 0.0000000 | 0.0000000 |
| EPHA3 | 3.1995957 | 1.3791463 | -1.2141141 | 0.0000010 | 0.0000068 |
| RNF150 | 2.3525357 | 0.8546147 | -1.4608705 | 0.0000000 | 0.0000002 |
| FAXDC2 | 3.2627409 | 1.5546269 | -1.0695161 | 0.0000000 | 0.0000000 |
| CPXM2 | 9.1529303 | 3.6456733 | -1.3280484 | 0.0000203 | 0.0000886 |
| REEP2 | 1.8811159 | 0.8055441 | -1.2235533 | 0.0000002 | 0.0000021 |
| PI16 | 4.3769959 | 1.3192413 | -1.7302325 | 0.0000146 | 0.0000665 |
| C7 | 17.2719802 | 6.2271047 | -1.4718001 | 0.0000006 | 0.0000042 |
| CD207 | 0.7850704 | 0.3517685 | -1.1581956 | 0.0000000 | 0.0000000 |
| FMO2 | 3.2545646 | 1.3769463 | -1.2409923 | 0.0000297 | 0.0001230 |
| LAMA2 | 3.6023048 | 1.7542446 | -1.0380703 | 0.0000000 | 0.0000000 |
| SCN4B | 1.0694879 | 0.4396882 | -1.2823674 | 0.0000000 | 0.0000000 |
| PPP1R3C | 4.3527253 | 1.5071329 | -1.5301124 | 0.0000000 | 0.0000000 |
| ARRDC5 | 0.7001612 | 0.3376785 | -1.0520370 | 0.0000031 | 0.0000174 |
| XG | 1.3483157 | 0.6404170 | -1.0740748 | 0.0000002 | 0.0000016 |
| PAGE1 | 0.5902665 | 2.2189189 | 1.9104185 | 0.0131524 | 0.0245516 |
| CCL23 | 2.0685868 | 0.5441703 | -1.9265155 | 0.0051646 | 0.0109760 |
| PDE1B | 0.8397580 | 0.4123188 | -1.0262133 | 0.0000000 | 0.0000000 |
| ALB | 69.4618132 | 0.8401678 | -6.3693988 | 0.0003160 | 0.0009652 |
| EFNA3 | 4.2591949 | 8.8055744 | 1.0478364 | 0.0000000 | 0.0000001 |
| SHISA3 | 4.3638800 | 1.2956221 | -1.7519664 | 0.0000375 | 0.0001505 |
| FCER1A | 2.0782277 | 0.6983744 | -1.5732810 | 0.0000000 | 0.0000000 |
| CCDC80 | 16.7644966 | 7.8293871 | -1.0984379 | 0.0000093 | 0.0000452 |
| PPP1R1A | 1.2301677 | 0.5951942 | -1.0474225 | 0.0012736 | 0.0032417 |
| LINGO3 | 1.2281613 | 0.5166263 | -1.2493071 | 0.0000000 | 0.0000000 |
| CCL21 | 64.4782105 | 29.6379957 | -1.1213638 | 0.0010427 | 0.0027273 |
| CHL1 | 0.9327492 | 0.3710123 | -1.3300222 | 0.0000000 | 0.0000000 |
| SERPINA3 | 0.9404230 | 0.4140273 | -1.1835839 | 0.0000116 | 0.0000547 |
| SLC26A9 | 5.3376647 | 2.6514531 | -1.0094255 | 0.0000225 | 0.0000968 |
| PRUNE2 | 8.8760767 | 3.1752090 | -1.4830706 | 0.0003279 | 0.0009983 |
| FOXP2 | 0.9217280 | 0.3750184 | -1.2973795 | 0.0000002 | 0.0000017 |
| LGALS9B | 2.0021759 | 0.9108606 | -1.1362666 | 0.0190625 | 0.0338330 |
| NEGR1 | 1.4933097 | 0.5692334 | -1.3914213 | 0.0000000 | 0.0000000 |
| CSTA | 37.1844587 | 9.1253980 | -2.0267404 | 0.0008422 | 0.0022624 |
| SCUBE2 | 1.0668352 | 0.5150073 | -1.0506726 | 0.0000001 | 0.0000008 |
| KCNE2 | 4.6595184 | 1.3673876 | -1.7687586 | 0.0000050 | 0.0000265 |
| RAB9B | 0.8924186 | 0.4276324 | -1.0613495 | 0.0000002 | 0.0000015 |
| NKX6-2 | 1.2090514 | 0.3876378 | -1.6410944 | 0.0008116 | 0.0021916 |
| CFL2 | 7.1153884 | 3.3445899 | -1.0891132 | 0.0000001 | 0.0000011 |
| TMOD1 | 2.6226423 | 1.1353742 | -1.2078532 | 0.0000043 | 0.0000231 |
| LGI2 | 2.5474986 | 1.2103678 | -1.0736358 | 0.0000028 | 0.0000162 |
| FCRL1 | 1.4329657 | 0.4185346 | -1.7755852 | 0.0042577 | 0.0092727 |
| CYP4B1 | 0.9081702 | 0.3538724 | -1.3597335 | 0.0000507 | 0.0001962 |
| PSD | 3.0258649 | 1.0615563 | -1.5111667 | 0.0001585 | 0.0005325 |
| SPINK5 | 14.5214065 | 3.7837840 | -1.9402795 | 0.0018527 | 0.0044796 |
| LMO3 | 0.6970562 | 0.3330685 | -1.0654562 | 0.0000187 | 0.0000828 |
| PDZD4 | 1.3276724 | 0.4357700 | -1.6072606 | 0.0000000 | 0.0000000 |
| SPEG | 2.1386672 | 0.7941525 | -1.4292241 | 0.0000516 | 0.0001992 |
| NFASC | 2.0352678 | 0.7790916 | -1.3853538 | 0.0000000 | 0.0000000 |
| TACR2 | 9.7734900 | 2.4860154 | -1.9750386 | 0.0183355 | 0.0327078 |
| KCNH3 | 0.9953070 | 0.3733939 | -1.4144431 | 0.0001378 | 0.0004693 |
| PSAPL1 | 5.1903933 | 1.2843242 | -2.0148345 | 0.0009756 | 0.0025689 |
| CDC6 | 6.1800669 | 14.6097625 | 1.2412384 | 0.0000000 | 0.0000000 |
| BEX4 | 11.8706729 | 5.4498766 | -1.1231063 | 0.0000000 | 0.0000000 |
| ADAMTSL3 | 1.1776110 | 0.4437863 | -1.4079261 | 0.0000000 | 0.0000000 |
| SIGLEC6 | 0.7432802 | 0.3314044 | -1.1653134 | 0.0000000 | 0.0000000 |
| CCKAR | 0.8594636 | 0.2264064 | -1.9245215 | 0.0023168 | 0.0054418 |
| ADH1A | 0.9815360 | 0.2862078 | -1.7779781 | 0.0059407 | 0.0124107 |
| LONRF2 | 0.7143427 | 0.2959829 | -1.2711023 | 0.0000000 | 0.0000004 |
| APOA1 | 60.8331263 | 24.7152553 | -1.2994553 | 0.0028843 | 0.0066007 |
| ELN | 18.2020735 | 8.5190486 | -1.0953386 | 0.0000000 | 0.0000000 |
| PLA2G5 | 0.8349138 | 0.3660568 | -1.1895597 | 0.0001226 | 0.0004243 |
| MAL | 26.4822852 | 3.1224775 | -3.0842645 | 0.0000000 | 0.0000000 |
| FGF10 | 1.2963899 | 0.5360709 | -1.2740039 | 0.0000128 | 0.0000593 |
| ALDH3A1 | 16.8303681 | 7.9443721 | -1.0830616 | 0.0005822 | 0.0016447 |
| MRGPRF | 13.3491360 | 4.9278898 | -1.4377045 | 0.0000046 | 0.0000245 |
| APOD | 67.0062643 | 33.1192226 | -1.0166272 | 0.0000000 | 0.0000005 |
| ARHGEF26 | 2.7488518 | 1.3168371 | -1.0617523 | 0.0000075 | 0.0000376 |
| LIMS2 | 5.9470804 | 2.4391662 | -1.2857935 | 0.0000000 | 0.0000002 |
| CACNA1C | 2.0762093 | 0.9625263 | -1.1090541 | 0.0000003 | 0.0000022 |
| ZNF750 | 2.5269183 | 0.6799020 | -1.8939802 | 0.0034678 | 0.0077826 |
| SFRP1 | 6.2710119 | 2.0911214 | -1.5844214 | 0.0000050 | 0.0000263 |
| SSC5D | 6.0099917 | 2.6545792 | -1.1788798 | 0.0000052 | 0.0000275 |
| FBXL22 | 1.8735908 | 0.7914667 | -1.2432052 | 0.0000059 | 0.0000306 |
| MICU3 | 0.7458124 | 0.3457079 | -1.1092591 | 0.0000000 | 0.0000000 |
| MS4A2 | 0.9872959 | 0.4094814 | -1.2696846 | 0.0000000 | 0.0000000 |
| CRISPLD1 | 2.8278816 | 1.4049019 | -1.0092523 | 0.0000000 | 0.0000003 |
| PRSS2 | 109.4435470 | 37.2169244 | -1.5561562 | 0.0155614 | 0.0283627 |
| TPSB2 | 12.4405980 | 5.9472448 | -1.0647625 | 0.0000000 | 0.0000000 |
| SLIT2 | 2.0537761 | 0.9209666 | -1.1570582 | 0.0000008 | 0.0000055 |
| SGCA | 3.2485702 | 1.0668114 | -1.6064998 | 0.0000000 | 0.0000001 |
| BOC | 3.3479914 | 1.2769790 | -1.3905610 | 0.0000000 | 0.0000000 |
| CLMP | 6.6989636 | 3.1941725 | -1.0684957 | 0.0000037 | 0.0000204 |
| MGP | 127.2543912 | 51.4020206 | -1.3078185 | 0.0000000 | 0.0000000 |
| RBMS3 | 1.9873303 | 0.9441242 | -1.0737830 | 0.0000000 | 0.0000000 |
| CDH2 | 1.8048205 | 0.6693773 | -1.4309639 | 0.0000121 | 0.0000567 |
| CNTN4 | 0.8917600 | 0.4326889 | -1.0433252 | 0.0000000 | 0.0000000 |
| GPA33 | 8.5637856 | 21.1443131 | 1.3039491 | 0.0000001 | 0.0000005 |
| CNTN1 | 1.6832965 | 0.7162036 | -1.2328477 | 0.0000002 | 0.0000017 |
| AC136428.1 | 3.7059427 | 1.7660676 | -1.0693000 | 0.0081753 | 0.0163222 |
| NOS2 | 4.4602922 | 11.5880863 | 1.3774322 | 0.0046922 | 0.0100922 |
| PDZRN4 | 1.1357491 | 0.3194571 | -1.8299499 | 0.0001929 | 0.0006309 |
| DPT | 7.0461184 | 2.8384507 | -1.3117251 | 0.0002213 | 0.0007088 |
| GRID1 | 0.7514127 | 0.3588828 | -1.0660928 | 0.0000001 | 0.0000009 |
| HOXA13 | 2.9821047 | 7.0811373 | 1.2476502 | 0.0000000 | 0.0000000 |
| SPESP1 | 1.1831519 | 0.5736843 | -1.0443065 | 0.0000000 | 0.0000000 |
| ARHGAP40 | 0.7744518 | 0.3333939 | -1.2159477 | 0.0031483 | 0.0071353 |
| COL23A1 | 0.9356854 | 0.4348523 | -1.1054980 | 0.0000001 | 0.0000013 |
| VSIG10L | 3.9343867 | 1.8054898 | -1.1237485 | 0.0275586 | 0.0463828 |
| MFAP4 | 91.0422269 | 38.0090044 | -1.2601946 | 0.0000000 | 0.0000000 |
| PDE7B | 1.4328097 | 0.6153846 | -1.2192867 | 0.0000000 | 0.0000000 |
| ABCC9 | 2.5380462 | 1.1504158 | -1.1415629 | 0.0000012 | 0.0000078 |
| HAS1 | 0.7550124 | 0.3499877 | -1.1091960 | 0.0004059 | 0.0012003 |
| ADH7 | 1.6543847 | 0.1973280 | -3.0676271 | 0.0233627 | 0.0403673 |
| AC119396.1 | 1.9984520 | 0.6215309 | -1.6849848 | 0.0000001 | 0.0000007 |
| ATP1A2 | 2.3945017 | 0.5633236 | -2.0876897 | 0.0000105 | 0.0000499 |
| TNS1 | 34.9986994 | 13.9531322 | -1.3267123 | 0.0000000 | 0.0000002 |
| APCDD1 | 14.4212927 | 6.3593408 | -1.1812514 | 0.0000267 | 0.0001122 |
| ARL4D | 3.2162996 | 1.4417427 | -1.1575881 | 0.0000000 | 0.0000004 |
| CPED1 | 5.4114948 | 2.1612009 | -1.3241940 | 0.0000000 | 0.0000001 |
| SCUBE1 | 0.8894354 | 0.4241994 | -1.0681471 | 0.0000018 | 0.0000110 |
| MSRB3 | 10.3868678 | 4.5363500 | -1.1951568 | 0.0000007 | 0.0000047 |
| ATOH8 | 1.0267510 | 0.4457778 | -1.2036895 | 0.0000000 | 0.0000000 |
| CHRNA3 | 1.6060565 | 0.5068531 | -1.6638831 | 0.0225685 | 0.0391716 |
| SMAD9 | 3.7435947 | 1.6858800 | -1.1509224 | 0.0000000 | 0.0000000 |
| LAMP5 | 2.6126764 | 1.2783167 | -1.0312832 | 0.0000817 | 0.0002979 |
| LYPD2 | 53.5319125 | 11.0852326 | -2.2717602 | 0.0112041 | 0.0214010 |
| CLDN10 | 1.6845927 | 0.3576045 | -2.2359631 | 0.0000142 | 0.0000650 |
| NLGN4Y | 0.7077298 | 0.3257770 | -1.1193139 | 0.0000004 | 0.0000033 |
| PDLIM3 | 13.3320163 | 5.9039084 | -1.1751528 | 0.0000022 | 0.0000128 |
| NBEA | 1.2568832 | 0.6025654 | -1.0606608 | 0.0000000 | 0.0000001 |
| PCP4L1 | 2.0339319 | 0.7914894 | -1.3616294 | 0.0000012 | 0.0000076 |
| SPARCL1 | 113.5254343 | 44.5162631 | -1.3506112 | 0.0000000 | 0.0000000 |
| SETBP1 | 2.4003088 | 1.0537618 | -1.1876712 | 0.0000000 | 0.0000000 |
| FGF2 | 1.7041478 | 0.7233785 | -1.2362279 | 0.0000000 | 0.0000000 |
| CCDC136 | 1.3564892 | 0.6599269 | -1.0394995 | 0.0001661 | 0.0005540 |
| LRRN4CL | 2.1806190 | 1.0721727 | -1.0242004 | 0.0000002 | 0.0000014 |
| DAPL1 | 2.5754324 | 1.2565880 | -1.0353029 | 0.0017090 | 0.0041793 |
| ASB5 | 0.8904738 | 0.2939046 | -1.5992251 | 0.0035210 | 0.0078817 |
| FNBP1 | 13.8527817 | 6.8336358 | -1.0194504 | 0.0000000 | 0.0000000 |
| SUSD4 | 2.1519950 | 1.0639813 | -1.0162019 | 0.0000383 | 0.0001534 |
| PLXNA4 | 0.9214374 | 0.3898621 | -1.2409223 | 0.0000005 | 0.0000034 |
| TENT5B | 4.8517438 | 2.0275376 | -1.2587747 | 0.0010427 | 0.0027273 |
| STUM | 0.8962496 | 0.2852719 | -1.6515627 | 0.0000001 | 0.0000006 |
| SERPINA5 | 4.4219278 | 1.9995705 | -1.1449853 | 0.0000004 | 0.0000029 |
| DPEP3 | 1.2630663 | 0.3979643 | -1.6662194 | 0.0000338 | 0.0001371 |
| CALCA | 7.7081402 | 0.1345143 | -5.8405510 | 0.0026046 | 0.0060426 |
| C6orf223 | 2.4398909 | 5.2560889 | 1.1071731 | 0.0000002 | 0.0000016 |
| HHIP | 0.8985037 | 0.4127805 | -1.1221497 | 0.0000001 | 0.0000006 |
| KCNH2 | 5.4037719 | 2.4940202 | -1.1154937 | 0.0007481 | 0.0020424 |
| CASQ2 | 3.6704727 | 0.9004740 | -2.0272094 | 0.0000000 | 0.0000003 |
| CD1C | 2.3523613 | 1.0887488 | -1.1114385 | 0.0000000 | 0.0000001 |
| HOXA10 | 2.8155994 | 5.7199053 | 1.0225492 | 0.0000000 | 0.0000000 |
| P2RX1 | 1.7138839 | 0.8294621 | -1.0470213 | 0.0000058 | 0.0000301 |
| BNC2 | 1.3655158 | 0.6365663 | -1.1010633 | 0.0000005 | 0.0000037 |
| PTGDS | 28.8877224 | 13.6939711 | -1.0769156 | 0.0000001 | 0.0000008 |
| AADAC | 9.3911994 | 4.4059064 | -1.0918706 | 0.0000065 | 0.0000332 |
| CACNA1H | 9.1002313 | 4.2371273 | -1.1028167 | 0.0000003 | 0.0000023 |
| GRIK5 | 1.1943670 | 0.4670575 | -1.3545741 | 0.0000010 | 0.0000065 |
| NPY1R | 0.8796641 | 0.2874157 | -1.6138139 | 0.0000000 | 0.0000002 |
| MRVI1 | 11.5409780 | 5.0238526 | -1.1998994 | 0.0000000 | 0.0000001 |
| SFRP5 | 3.5913039 | 1.1757575 | -1.6109172 | 0.0000000 | 0.0000000 |
| PRSS1 | 36.7572797 | 7.8976823 | -2.2185288 | 0.0112058 | 0.0214010 |
| DZIP1 | 1.4829955 | 0.7315167 | -1.0195516 | 0.0000000 | 0.0000000 |
| CTNND2 | 0.7931426 | 0.2540091 | -1.6427000 | 0.0000000 | 0.0000000 |
| BVES | 2.2518580 | 0.8914524 | -1.3368862 | 0.0000000 | 0.0000000 |
| NPR3 | 0.7222279 | 0.3268880 | -1.1436576 | 0.0000142 | 0.0000651 |
| RBPMS2 | 12.4274551 | 3.9735434 | -1.6450329 | 0.0000003 | 0.0000021 |
| AOC3 | 17.6400584 | 7.1975965 | -1.2932682 | 0.0000000 | 0.0000003 |
| HAS3 | 3.8768634 | 1.8525888 | -1.0653472 | 0.0161272 | 0.0292465 |
| TCEAL2 | 2.9032257 | 1.3113903 | -1.1465597 | 0.0000248 | 0.0001053 |
| ACTC1 | 2.1623384 | 0.8773234 | -1.3014117 | 0.0000418 | 0.0001659 |
| FAM13C | 0.8240444 | 0.3933721 | -1.0668274 | 0.0000000 | 0.0000000 |
| SPX | 1.5617174 | 0.0475336 | -5.0380409 | 0.0000005 | 0.0000039 |
| JCHAIN | 373.9198589 | 156.5610984 | -1.2560033 | 0.0007560 | 0.0020599 |
| SGCD | 2.8590823 | 1.3788375 | -1.0520996 | 0.0000011 | 0.0000070 |
| HOXA9 | 0.6270455 | 2.7923278 | 2.1548263 | 0.0000000 | 0.0000000 |
| COL22A1 | 1.4938855 | 0.7289339 | -1.0352098 | 0.0000325 | 0.0001326 |
| CCKBR | 1.1107059 | 0.3615577 | -1.6191789 | 0.0008422 | 0.0022624 |
| CPA3 | 13.1728520 | 5.7702959 | -1.1908505 | 0.0000000 | 0.0000000 |
| PRKCB | 3.5645788 | 1.6142187 | -1.1428956 | 0.0000022 | 0.0000130 |
| SCARA5 | 2.1486733 | 0.7823768 | -1.4575107 | 0.0000000 | 0.0000000 |
| TNN | 0.8589020 | 0.3982782 | -1.1087170 | 0.0000016 | 0.0000100 |
| DPYSL3 | 35.1871387 | 16.2879588 | -1.1112424 | 0.0000002 | 0.0000016 |
| METTL24 | 1.5165250 | 0.5708768 | -1.4095180 | 0.0000000 | 0.0000005 |
| TMEM35A | 1.1755999 | 0.3518542 | -1.7403476 | 0.0000015 | 0.0000096 |
| RET | 1.7522189 | 0.7446414 | -1.2345653 | 0.0000017 | 0.0000107 |
| MOXD1 | 4.7605946 | 2.3403888 | -1.0243936 | 0.0000000 | 0.0000002 |
| FLNA | 257.8719013 | 117.6956920 | -1.1315931 | 0.0000103 | 0.0000492 |
| MSMB | 118.5704535 | 39.5477797 | -1.5840759 | 0.0001644 | 0.0005491 |
| PRELP | 17.1011434 | 6.5470287 | -1.3851806 | 0.0000002 | 0.0000019 |
| ANK2 | 1.3648071 | 0.4937235 | -1.4669219 | 0.0000000 | 0.0000000 |
| ZNF334 | 0.8126378 | 0.3545892 | -1.1964639 | 0.0000000 | 0.0000000 |
| RDH12 | 1.1654698 | 0.4493596 | -1.3749692 | 0.0000061 | 0.0000314 |
| ABI3BP | 5.7143724 | 2.1133496 | -1.4350636 | 0.0000001 | 0.0000010 |
| FEZF1 | 0.8551040 | 1.7132806 | 1.0025897 | 0.0000010 | 0.0000065 |
| CCL19 | 26.5754963 | 9.2683350 | -1.5197145 | 0.0000011 | 0.0000072 |
| AOX1 | 1.9847388 | 0.6900610 | -1.5241534 | 0.0000008 | 0.0000053 |
| FLRT2 | 0.7300420 | 0.2970311 | -1.2973655 | 0.0000000 | 0.0000002 |
| BCHE | 1.4091499 | 0.5120203 | -1.4605522 | 0.0000006 | 0.0000043 |
| PCLO | 1.0292991 | 0.4537669 | -1.1816390 | 0.0000059 | 0.0000302 |
| DMD | 2.1679447 | 0.9785389 | -1.1476269 | 0.0000000 | 0.0000002 |
| GLRB | 0.8466723 | 0.3943233 | -1.1024248 | 0.0000000 | 0.0000005 |
| SCNN1B | 2.0575332 | 0.4373695 | -2.2339913 | 0.0000009 | 0.0000062 |
| SYNM | 34.2274687 | 8.4786393 | -2.0132499 | 0.0000007 | 0.0000048 |
| COL4A6 | 1.2421746 | 0.3819299 | -1.7014881 | 0.0000000 | 0.0000005 |
| ADCY5 | 4.2223236 | 1.4114985 | -1.5808096 | 0.0000000 | 0.0000000 |
| NTN1 | 7.1583496 | 2.8086039 | -1.3497738 | 0.0000000 | 0.0000000 |
| ABCA8 | 1.5662382 | 0.4533625 | -1.7885667 | 0.0000000 | 0.0000000 |
| S100A1 | 1.6989929 | 0.8086508 | -1.0710911 | 0.0146963 | 0.0269998 |
| LEFTY1 | 2.8350973 | 11.1505663 | 1.9756468 | 0.0005238 | 0.0015024 |
| SOX8 | 2.1699089 | 0.8789648 | -1.3037572 | 0.0015306 | 0.0037973 |
| NXPH3 | 1.1481672 | 0.4047850 | -1.5041050 | 0.0000028 | 0.0000159 |
| SYN2 | 1.0439224 | 0.3905415 | -1.4184665 | 0.0000000 | 0.0000000 |
| CLEC3B | 5.0160410 | 2.3473532 | -1.0955142 | 0.0000000 | 0.0000000 |
| FGF7 | 4.4226115 | 2.1688655 | -1.0279580 | 0.0000006 | 0.0000044 |
| C1QTNF3 | 3.0039311 | 1.3769528 | -1.1253726 | 0.0031002 | 0.0070363 |
| GPR20 | 0.9189423 | 0.4230089 | -1.1192861 | 0.0000000 | 0.0000000 |
| WNT2B | 1.4847850 | 0.6961410 | -1.0928026 | 0.0000001 | 0.0000011 |
| FAM129A | 13.4374822 | 6.3953830 | -1.0711602 | 0.0000330 | 0.0001346 |
| NRXN2 | 0.9138546 | 0.4148212 | -1.1394750 | 0.0000000 | 0.0000005 |
| GFRA1 | 2.4310410 | 0.8171149 | -1.5729633 | 0.0000000 | 0.0000000 |
| FAM129C | 0.7679668 | 0.2479188 | -1.6311760 | 0.0001362 | 0.0004647 |
| CNN1 | 145.2829974 | 34.2142965 | -2.0861947 | 0.0001936 | 0.0006327 |
| SHISAL2A | 0.9954607 | 0.4538218 | -1.1332384 | 0.0000072 | 0.0000362 |
| FAM180A | 1.2213439 | 0.5919954 | -1.0448116 | 0.0000094 | 0.0000455 |
| TNFAIP8L3 | 1.9758747 | 0.8761764 | -1.1731981 | 0.0000000 | 0.0000000 |
| GNG4 | 3.8871319 | 1.9329150 | -1.0079279 | 0.0014610 | 0.0036504 |
| STON1 | 2.8226843 | 1.3063739 | -1.1114999 | 0.0000002 | 0.0000015 |
| SOX2 | 9.7878341 | 4.4957633 | -1.1224236 | 0.0000012 | 0.0000077 |
| HAND2 | 6.3940363 | 1.8655600 | -1.7771182 | 0.0068410 | 0.0140253 |
| CHRDL1 | 5.1281493 | 1.8218753 | -1.4930140 | 0.0000004 | 0.0000028 |
| XCR1 | 0.7811938 | 0.3023164 | -1.3696214 | 0.0015004 | 0.0037326 |
| CPA2 | 52.6024981 | 1.3485420 | -5.2856590 | 0.0146618 | 0.0269441 |
| GREM2 | 3.3478630 | 1.2199183 | -1.4564559 | 0.0000000 | 0.0000000 |
| ANKRD35 | 1.2010556 | 0.5081429 | -1.2409968 | 0.0000000 | 0.0000000 |
| GNAO1 | 2.9233778 | 1.0278378 | -1.5080237 | 0.0000000 | 0.0000000 |
| C1QTNF7 | 1.2352260 | 0.3908721 | -1.6600065 | 0.0000000 | 0.0000000 |
| FCRL3 | 1.1989530 | 0.5209204 | -1.2026402 | 0.0252690 | 0.0431198 |
| ACTL8 | 1.3327187 | 5.2476998 | 1.9773129 | 0.0002259 | 0.0007221 |
| ADRB2 | 0.8901724 | 0.3718590 | -1.2593292 | 0.0000000 | 0.0000000 |
| TUBAL3 | 0.5179373 | 1.2277759 | 1.2451980 | 0.0000275 | 0.0001150 |
| CALD1 | 85.1927609 | 40.3829110 | -1.0769859 | 0.0000136 | 0.0000627 |
| PHYHIPL | 1.1819184 | 0.5564828 | -1.0867214 | 0.0000062 | 0.0000319 |
| MORN5 | 1.2905609 | 0.3509697 | -1.8785798 | 0.0068741 | 0.0140773 |
| PPP1R14A | 14.1635298 | 5.6460463 | -1.3268680 | 0.0000000 | 0.0000002 |
| DACT3 | 4.1431744 | 1.5629567 | -1.4064588 | 0.0000004 | 0.0000029 |
| GAPT | 1.5479123 | 0.6869686 | -1.1720077 | 0.0000000 | 0.0000001 |
| FGA | 7.2922632 | 1.3632231 | -2.4193450 | 0.0002949 | 0.0009085 |
| PENK | 0.8813592 | 0.1816844 | -2.2782954 | 0.0101281 | 0.0196237 |
| MAMDC2 | 2.4237987 | 0.6541544 | -1.8895667 | 0.0000000 | 0.0000000 |
| MS4A1 | 6.5168595 | 2.8352408 | -1.2007056 | 0.0009703 | 0.0025570 |
| JPH2 | 5.4026094 | 1.6567428 | -1.7053067 | 0.0000002 | 0.0000019 |
| GNG7 | 2.2586776 | 1.0720822 | -1.0750629 | 0.0000000 | 0.0000000 |
| FRZB | 12.0856497 | 4.6330372 | -1.3832649 | 0.0000000 | 0.0000000 |
| OGN | 19.2372389 | 4.9051699 | -1.9715267 | 0.0000003 | 0.0000027 |
| DLX5 | 1.0160758 | 0.4754946 | -1.0955070 | 0.0146179 | 0.0268869 |
| CR2 | 7.8961654 | 3.4401713 | -1.1986718 | 0.0023614 | 0.0055382 |
| HTR1B | 1.2459289 | 0.5715131 | -1.1243633 | 0.0000033 | 0.0000186 |
| BARX1 | 26.6606291 | 9.4231954 | -1.5004226 | 0.0000087 | 0.0000428 |
| JAM2 | 3.6333512 | 1.6487219 | -1.1399528 | 0.0000000 | 0.0000000 |
| BHMT2 | 1.1425547 | 0.4308623 | -1.4069646 | 0.0000000 | 0.0000000 |
| PLA2G1B | 34.8387859 | 0.6104065 | -5.8347802 | 0.0261575 | 0.0443609 |
| NPTX1 | 1.7230492 | 0.3056991 | -2.4947798 | 0.0000001 | 0.0000008 |
| CCR7 | 4.8462036 | 2.2583331 | -1.1015967 | 0.0000263 | 0.0001106 |
| MGAM | 1.0240497 | 0.4514235 | -1.1817322 | 0.0299519 | 0.0497822 |
| NIPAL4 | 0.7373489 | 0.2793725 | -1.4001573 | 0.0000002 | 0.0000014 |
| GHR | 0.9700184 | 0.4459785 | -1.1210379 | 0.0000000 | 0.0000000 |
| SERPINA6 | 3.4014178 | 1.2946932 | -1.3935259 | 0.0009853 | 0.0025916 |
| ARHGEF25 | 3.9864970 | 1.9801613 | -1.0095036 | 0.0000007 | 0.0000052 |
| HOXA11 | 1.0667664 | 3.1530184 | 1.5634893 | 0.0000000 | 0.0000000 |
| TUBB2B | 4.6601520 | 1.8666267 | -1.3199435 | 0.0000078 | 0.0000388 |
| NEXN | 12.3542415 | 5.5117963 | -1.1644120 | 0.0000162 | 0.0000731 |
| RUNX1T1 | 1.0909034 | 0.4655535 | -1.2285045 | 0.0000000 | 0.0000004 |
| MEOX2 | 1.7894938 | 0.8300392 | -1.1083002 | 0.0000921 | 0.0003306 |
| AGTR1 | 0.9153726 | 0.4346481 | -1.0745114 | 0.0002306 | 0.0007352 |
| RCAN2 | 9.9056986 | 4.0299164 | -1.2975088 | 0.0000000 | 0.0000000 |
| GDNF | 0.8882577 | 0.3198588 | -1.4735432 | 0.0000002 | 0.0000020 |
| TP63 | 1.3639109 | 0.3176959 | -2.1020310 | 0.0017930 | 0.0043495 |
| ATP1B2 | 1.6704026 | 0.8345615 | -1.0011055 | 0.0000000 | 0.0000000 |
| TSPAN32 | 0.7596506 | 0.3411009 | -1.1551376 | 0.0000896 | 0.0003228 |
| KCNK3 | 1.8899107 | 0.7917291 | -1.2552392 | 0.0000000 | 0.0000002 |
| PTGER3 | 1.2659209 | 0.6045359 | -1.0662873 | 0.0000097 | 0.0000469 |
| CYP1B1 | 7.9837977 | 3.9544369 | -1.0136029 | 0.0000021 | 0.0000123 |
| CPM | 9.2580851 | 4.2424929 | -1.1258016 | 0.0000024 | 0.0000142 |
| CNR1 | 0.9839378 | 0.4821267 | -1.0291548 | 0.0000000 | 0.0000002 |
| CRYAB | 11.0324524 | 4.3824545 | -1.3319425 | 0.0000007 | 0.0000048 |
| SCRG1 | 2.0653383 | 0.6268031 | -1.7202940 | 0.0001155 | 0.0004019 |
| TMEM100 | 1.6278971 | 0.5750260 | -1.5013104 | 0.0000000 | 0.0000000 |
| PPP1R12B | 15.7904699 | 6.0853637 | -1.3756387 | 0.0008364 | 0.0022486 |
| BEND5 | 0.9856124 | 0.4180273 | -1.2374231 | 0.0000000 | 0.0000000 |
| NXPH4 | 1.8439579 | 4.6476058 | 1.3336820 | 0.0000172 | 0.0000769 |
| ACTG2 | 331.5969572 | 80.5993197 | -2.0405912 | 0.0003428 | 0.0010363 |
| DDR2 | 7.6155710 | 3.7968444 | -1.0041514 | 0.0000020 | 0.0000118 |
| SAMD11 | 2.3510038 | 1.0737881 | -1.1305676 | 0.0000000 | 0.0000000 |
| GPR27 | 1.6542080 | 0.6376109 | -1.3753924 | 0.0000000 | 0.0000000 |
| HLF | 1.4417304 | 0.5974121 | -1.2710030 | 0.0000003 | 0.0000021 |
| NAT2 | 1.1165549 | 2.3517596 | 1.0746864 | 0.0064610 | 0.0133653 |
| PRRG3 | 1.8611683 | 0.7365517 | -1.3373498 | 0.0010462 | 0.0027333 |
| CX3CR1 | 1.2516246 | 0.4834280 | -1.3724290 | 0.0000004 | 0.0000033 |
| ITIH5 | 2.9031768 | 1.3029298 | -1.1558731 | 0.0000000 | 0.0000000 |
| THBS4 | 20.3757710 | 6.8551679 | -1.5715908 | 0.0000041 | 0.0000224 |
| PTGER1 | 3.0319899 | 1.4467528 | -1.0674466 | 0.0000000 | 0.0000001 |
| PI15 | 2.4652187 | 1.2268684 | -1.0067351 | 0.0262293 | 0.0444647 |
| SLC2A4 | 2.7556772 | 0.9264459 | -1.5726283 | 0.0030330 | 0.0069035 |
| SYNPO2 | 35.6607567 | 10.3834974 | -1.7800449 | 0.0000002 | 0.0000017 |
| FLNC | 39.4660384 | 11.4112284 | -1.7901576 | 0.0007325 | 0.0020034 |
| FXYD6 | 4.5325943 | 2.1301769 | -1.0893638 | 0.0000000 | 0.0000003 |
| FHL1 | 29.0035885 | 9.3320297 | -1.6359686 | 0.0000000 | 0.0000001 |
| KCNMB1 | 5.9768887 | 2.0009498 | -1.5787097 | 0.0000126 | 0.0000585 |
| SYNC | 2.1831821 | 0.9619027 | -1.1824696 | 0.0002837 | 0.0008776 |
| PTGIS | 11.4511473 | 5.0360418 | -1.1851300 | 0.0000002 | 0.0000018 |
| OMD | 3.9482583 | 1.5885199 | -1.3135332 | 0.0000243 | 0.0001035 |
| C2orf40 | 8.8638902 | 1.9508828 | -2.1838129 | 0.0000009 | 0.0000059 |
| CLIP3 | 10.7168149 | 4.7171120 | -1.1839004 | 0.0000000 | 0.0000000 |
| P2RY14 | 2.6907665 | 1.2536660 | -1.1018642 | 0.0000000 | 0.0000001 |
| PRICKLE2 | 2.7369489 | 1.2135961 | -1.1732802 | 0.0000000 | 0.0000000 |
| MAPK10 | 0.7409344 | 0.3382788 | -1.1311333 | 0.0000000 | 0.0000000 |
| ANGPTL1 | 3.7695393 | 1.1110988 | -1.7624011 | 0.0000017 | 0.0000104 |
| RPL22L1 | 17.7705746 | 35.7482209 | 1.0083811 | 0.0000002 | 0.0000021 |
| SPDYC | 0.8953240 | 2.0377665 | 1.1865071 | 0.0000086 | 0.0000425 |
| FDCSP | 41.2280687 | 12.2777589 | -1.7475796 | 0.0000477 | 0.0001859 |
| KRT5 | 46.0395790 | 6.5490788 | -2.8135107 | 0.0238552 | 0.0410566 |
| C2CD4A | 3.7256026 | 7.8795872 | 1.0806463 | 0.0000014 | 0.0000089 |
| SEMA3E | 1.5277759 | 0.6767489 | -1.1747404 | 0.0000000 | 0.0000001 |
| PRR29 | 0.7734144 | 0.3472517 | -1.1552601 | 0.0000000 | 0.0000003 |
| CDO1 | 1.0964158 | 0.4475846 | -1.2925627 | 0.0000032 | 0.0000178 |
| NAT8L | 0.8650239 | 0.4056920 | -1.0923553 | 0.0000119 | 0.0000557 |
| SSTR5 | 0.5371059 | 1.1570878 | 1.1072199 | 0.0022268 | 0.0052546 |
| FOXF1 | 12.7267066 | 6.1487207 | -1.0495009 | 0.0000000 | 0.0000000 |
| ACKR1 | 13.2756933 | 5.1628096 | -1.3625589 | 0.0000000 | 0.0000000 |
| PTCH2 | 1.8735735 | 0.7427967 | -1.3347533 | 0.0000000 | 0.0000003 |
| CXCL17 | 110.5569839 | 36.2042812 | -1.6105580 | 0.0000028 | 0.0000162 |
| GALNT17 | 0.7910380 | 0.3858369 | -1.0357558 | 0.0000000 | 0.0000000 |
| DAAM2 | 4.4590903 | 2.0467678 | -1.1234020 | 0.0000000 | 0.0000000 |
| ADAMTS8 | 2.2172783 | 0.9104718 | -1.2841037 | 0.0000007 | 0.0000051 |
| ZCCHC24 | 11.0687998 | 5.4326901 | -1.0267601 | 0.0000000 | 0.0000001 |
| RNF180 | 0.9307187 | 0.4612114 | -1.0129171 | 0.0000000 | 0.0000000 |
| PGA3 | 45.5733103 | 6.9857896 | -2.7056941 | 0.0004930 | 0.0014263 |
| SMOC2 | 27.3712930 | 11.9314875 | -1.1978897 | 0.0000030 | 0.0000171 |
| CYP17A1 | 4.0482495 | 0.9634125 | -2.0710727 | 0.0006183 | 0.0017314 |
| CADM3 | 1.2096371 | 0.4642240 | -1.3816811 | 0.0000002 | 0.0000018 |
| PGM5 | 7.3324627 | 2.1343330 | -1.7805125 | 0.0000021 | 0.0000123 |
| C8G | 2.0694737 | 6.7367093 | 1.7027801 | 0.0024304 | 0.0056768 |
| NHSL2 | 1.2701824 | 0.6005424 | -1.0806977 | 0.0000000 | 0.0000001 |
| SHISAL1 | 1.8030728 | 0.7069446 | -1.3507886 | 0.0000031 | 0.0000174 |
| MAP6 | 1.1690945 | 0.4657016 | -1.3279139 | 0.0000000 | 0.0000000 |
| ERP27 | 5.9415946 | 1.1240366 | -2.4021611 | 0.0002281 | 0.0007280 |
| ZIC5 | 0.5249362 | 1.2843753 | 1.2908529 | 0.0000000 | 0.0000001 |
| EPHA7 | 1.6189081 | 0.5591572 | -1.5336953 | 0.0000003 | 0.0000024 |
| PPP1R1B | 29.7522185 | 81.8123905 | 1.4593221 | 0.0000000 | 0.0000001 |
| SLC9A4 | 2.7947069 | 1.1877867 | -1.2344212 | 0.0000974 | 0.0003470 |
| NCCRP1 | 8.5376365 | 2.7454061 | -1.6368172 | 0.0000045 | 0.0000240 |
| IL10 | 1.1886049 | 0.4106143 | -1.5334135 | 0.0052266 | 0.0110817 |
| SEMG1 | 0.7778818 | 2.3149688 | 1.5733699 | 0.0002486 | 0.0007832 |
| PLIN4 | 5.7358779 | 1.8619181 | -1.6232247 | 0.0000628 | 0.0002359 |
| AVIL | 2.0109834 | 0.9143189 | -1.1371318 | 0.0063141 | 0.0130936 |
| EML1 | 3.6110486 | 1.6910568 | -1.0944927 | 0.0000000 | 0.0000000 |
| MYOCD | 3.1258081 | 0.9972984 | -1.6481321 | 0.0000001 | 0.0000008 |
| ASCL1 | 7.7153772 | 0.8923020 | -3.1121327 | 0.0005074 | 0.0014613 |
| PRKAA2 | 1.2755234 | 0.5885965 | -1.1157385 | 0.0000000 | 0.0000000 |
| PRSS27 | 1.4007690 | 0.3859052 | -1.8599008 | 0.0259057 | 0.0439987 |
| PTGFR | 1.0142863 | 0.4843454 | -1.0663567 | 0.0000002 | 0.0000016 |
| SPIB | 5.4681181 | 2.0662288 | -1.4040444 | 0.0098913 | 0.0191944 |
| SCARA3 | 8.5356667 | 3.9955660 | -1.0951039 | 0.0000000 | 0.0000000 |
| FAM198A | 1.0448137 | 0.4718157 | -1.1469504 | 0.0000000 | 0.0000000 |
| PTN | 6.6114888 | 3.0654416 | -1.1088802 | 0.0000000 | 0.0000002 |
| NAP1L3 | 0.8804484 | 0.4281160 | -1.0402368 | 0.0000002 | 0.0000019 |
| ACTA2 | 190.4412100 | 82.8231849 | -1.2012391 | 0.0000219 | 0.0000943 |
| DNER | 2.2015652 | 0.4857178 | -2.1803393 | 0.0002396 | 0.0007590 |
| INMT | 3.7625035 | 1.6462403 | -1.1925180 | 0.0000000 | 0.0000003 |
| TTLL7 | 1.4363125 | 0.7149506 | -1.0064542 | 0.0000014 | 0.0000090 |
| GPR87 | 1.4684352 | 0.5163111 | -1.5079671 | 0.0000403 | 0.0001608 |
| C14orf132 | 3.3038927 | 1.3343083 | -1.3080747 | 0.0000000 | 0.0000000 |
| SOX15 | 3.0442112 | 1.3047297 | -1.2223175 | 0.0000006 | 0.0000045 |
| SVEP1 | 2.4537216 | 1.0797159 | -1.1843198 | 0.0000012 | 0.0000078 |
| CYS1 | 3.5885453 | 1.5581011 | -1.2036103 | 0.0000034 | 0.0000190 |
| RTL1 | 1.1935915 | 0.0935102 | -3.6740419 | 0.0011978 | 0.0030709 |
