# Supplementary material for: Identification of a Gene Prognostic Model of Gastric Cancer Based on Analysis of Tumor Mutation Burden
Source: Pathol Oncol Res. 2021 Sep 10;27:1609852. doi: 10.3389/pore.2021.1609852 (PMC8460769; doi:10.3389/pore.2021.1609852)
Supplement: Supplementary file 2 [file table2.docx]

| Table S2 Prognosis-related DEGs identified by univariate Cox regression analysis. | | | | |
| --- | --- | --- | --- | --- |
| Gene | HR | HR.95L | HR.95H | p value |
| PDE3A | 1.053893734 | 1.021055163 | 1.087788439 | 0.001153697 |
| GC | 1.005483881 | 1.000560643 | 1.010431344 | 0.028979156 |
| ADGRD1 | 1.246055118 | 1.030631797 | 1.506506361 | 0.023115708 |
| SCGB3A1 | 1.001550207 | 1.000543597 | 1.00255783 | 0.00253428 |
| TMTC1 | 1.020631951 | 1.002769885 | 1.03881219 | 0.023388981 |
| SELP | 1.062396651 | 1.013490852 | 1.113662391 | 0.011826228 |
| ITGA8 | 1.021439038 | 1.001190479 | 1.042097113 | 0.037854445 |
| TCEAL7 | 1.140519825 | 1.044474053 | 1.245397593 | 0.003395815 |
| CHRDL2 | 1.00940178 | 1.002393833 | 1.016458721 | 0.008473259 |
| HSPB7 | 1.008139209 | 1.000634719 | 1.015699981 | 0.033469251 |
| MPDZ | 1.15086618 | 1.043182038 | 1.269666189 | 0.005056629 |
| KCNMA1 | 1.029943259 | 1.001304077 | 1.059401576 | 0.040311798 |
| MAP1B | 1.024670853 | 1.002917305 | 1.046896241 | 0.026011729 |
| CHRM2 | 1.091722716 | 1.011894139 | 1.177848988 | 0.023502783 |
| C8orf88 | 1.049582107 | 1.00120553 | 1.10029616 | 0.044430497 |
| GPRASP1 | 1.119059555 | 1.012548072 | 1.236775145 | 0.027501436 |
| SPON1 | 1.009539797 | 1.001587558 | 1.017555174 | 0.018617258 |
| CH25H | 1.063973808 | 1.002630025 | 1.12907078 | 0.040692119 |
| ROR2 | 1.059801414 | 1.013275587 | 1.108463533 | 0.011221013 |
| NRXN3 | 1.121310009 | 1.030855802 | 1.219701275 | 0.007627651 |
| CILP | 1.031717547 | 1.001804736 | 1.062523522 | 0.037518205 |
| HSPB8 | 1.006226609 | 1.000042133 | 1.012449331 | 0.048454773 |
| UPK1B | 1.023785879 | 1.011471679 | 1.03625 | 0.000140425 |
| EPHA3 | 1.05644047 | 1.001368297 | 1.114541443 | 0.044428896 |
| RNF150 | 1.062887879 | 1.007796051 | 1.120991338 | 0.024707858 |
| FAXDC2 | 1.055309903 | 1.002137111 | 1.111304011 | 0.041261098 |
| C7 | 1.005845183 | 1.000201255 | 1.011520958 | 0.042350657 |
| LAMA2 | 1.075092712 | 1.016687636 | 1.13685295 | 0.011063586 |
| XG | 1.12601668 | 1.044024597 | 1.214447981 | 0.002091916 |
| PDE1B | 1.515503312 | 1.225866426 | 1.873573043 | 0.000122122 |
| EFNA3 | 0.968171303 | 0.940718225 | 0.996425546 | 0.027527862 |
| CCL21 | 1.000770011 | 1.000114659 | 1.001425792 | 0.021278361 |
| PRUNE2 | 1.014292425 | 1.001004004 | 1.027757252 | 0.034935591 |
| FOXP2 | 1.197966374 | 1.035015099 | 1.386572462 | 0.015464387 |
| TMOD1 | 1.073137728 | 1.016187354 | 1.133279783 | 0.011176396 |
| LMO3 | 1.387432382 | 1.065813286 | 1.80610304 | 0.014946566 |
| TACR2 | 1.009493127 | 1.000787486 | 1.018274497 | 0.032508918 |
| CDC6 | 1.003888464 | 1.00068598 | 1.007101197 | 0.017284052 |
| BEX4 | 1.021900022 | 1.004930142 | 1.039156467 | 0.011225928 |
| SIGLEC6 | 1.336497604 | 1.075106296 | 1.661441155 | 0.008997171 |
| PLA2G5 | 1.199647101 | 1.108016885 | 1.298854905 | 7.12E-06 |
| FGF10 | 1.103752507 | 1.009521549 | 1.206779189 | 0.030151327 |
| APOD | 1.002137622 | 1.000394279 | 1.003884002 | 0.016229708 |
| MICU3 | 1.435836695 | 1.123885181 | 1.834375121 | 0.003798272 |
| SLIT2 | 1.077922472 | 1.006054684 | 1.154924155 | 0.033053523 |
| SGCA | 1.034130075 | 1.000198488 | 1.069212786 | 0.048652585 |
| MGP | 1.001588209 | 1.000287015 | 1.002891097 | 0.016727999 |
| RBMS3 | 1.226408118 | 1.099456149 | 1.368018973 | 0.000251626 |
| CDH2 | 1.06006329 | 1.003387705 | 1.119940151 | 0.037470507 |
| CNTN4 | 1.330633681 | 1.020683873 | 1.734705563 | 0.034748338 |
| CNTN1 | 1.037375998 | 1.004256307 | 1.071587954 | 0.026655935 |
| MFAP4 | 1.002512992 | 1.00026412 | 1.00476692 | 0.02849217 |
| PDE7B | 1.160874738 | 1.046419866 | 1.287848407 | 0.004851355 |
| ABCC9 | 1.131514288 | 1.057870119 | 1.210285233 | 0.00032023 |
| CPED1 | 1.032243596 | 1.003701099 | 1.061597762 | 0.026542207 |
| MSRB3 | 1.016209733 | 1.001408725 | 1.031229503 | 0.031712465 |
| CHRNA3 | 1.074955769 | 1.01663333 | 1.136624061 | 0.011098653 |
| LAMP5 | 1.041804961 | 1.006607977 | 1.078232641 | 0.019514007 |
| NLGN4Y | 1.340278083 | 1.011770306 | 1.775447775 | 0.04119807 |
| SPARCL1 | 1.001866734 | 1.000443573 | 1.00329192 | 0.010128305 |
| SETBP1 | 1.114771911 | 1.017901854 | 1.220860742 | 0.019154082 |
| ASB5 | 1.111745232 | 1.005370835 | 1.229374692 | 0.038984644 |
| SERPINA5 | 1.02380053 | 1.000334631 | 1.047816893 | 0.046784607 |
| CASQ2 | 1.02341895 | 1.003394532 | 1.043842989 | 0.021670758 |
| BNC2 | 1.156287748 | 1.00991304 | 1.323877703 | 0.035482858 |
| MRVI1 | 1.016178379 | 1.000719694 | 1.031875862 | 0.040174708 |
| NPR3 | 1.08414188 | 1.020734191 | 1.151488435 | 0.008604604 |
| CPA3 | 1.016745446 | 1.005680938 | 1.027931686 | 0.002932961 |
| DPYSL3 | 1.006002247 | 1.001215823 | 1.010811552 | 0.013920185 |
| RDH12 | 1.058402714 | 1.005690952 | 1.113877284 | 0.029429599 |
| FLRT2 | 1.372700263 | 1.102315925 | 1.709406505 | 0.004650163 |
| GLRB | 1.307189461 | 1.042677983 | 1.638803459 | 0.020218665 |
| COL4A6 | 1.054158688 | 1.000920947 | 1.110228077 | 0.046067005 |
| ABCA8 | 1.114576194 | 1.004032843 | 1.237290294 | 0.041801904 |
| FGF7 | 1.048333558 | 1.010292474 | 1.087807023 | 0.012316341 |
| TNFAIP8L3 | 1.041227241 | 1.006715423 | 1.076922178 | 0.018816825 |
| CHRDL1 | 1.024911182 | 1.000190419 | 1.050242945 | 0.048239956 |
| FRZB | 1.019063743 | 1.0075008 | 1.030759391 | 0.001180937 |
| GHR | 1.250947759 | 1.067172393 | 1.46637067 | 0.005746226 |
| RUNX1T1 | 1.186676958 | 1.014657528 | 1.387859611 | 0.032187405 |
| MEOX2 | 1.087535071 | 1.022649211 | 1.156537861 | 0.00750583 |
| RCAN2 | 1.010974469 | 1.001475535 | 1.020563499 | 0.023446126 |
| ATP1B2 | 1.131262493 | 1.01843504 | 1.25658955 | 0.021407052 |
| CYP1B1 | 1.035540887 | 1.017205275 | 1.054207006 | 0.000127364 |
| CRYAB | 1.015309474 | 1.002196566 | 1.028593954 | 0.021975749 |
| SCRG1 | 1.071021691 | 1.020972542 | 1.123524302 | 0.004954213 |
| TMEM100 | 1.099422075 | 1.015109804 | 1.190737095 | 0.01989324 |
| BEND5 | 1.220928937 | 1.030351971 | 1.446755585 | 0.021150989 |
| DDR2 | 1.036750162 | 1.009641103 | 1.064587105 | 0.007591476 |
| CX3CR1 | 1.07894769 | 1.000273971 | 1.163809268 | 0.049176835 |
| ITIH5 | 1.070902559 | 1.006356406 | 1.139588602 | 0.030793152 |
| FXYD6 | 1.047524626 | 1.00867413 | 1.087871503 | 0.016046023 |
| OMD | 1.044236438 | 1.009693832 | 1.07996078 | 0.011667144 |
| C2orf40 | 1.007483091 | 1.000444854 | 1.014570844 | 0.037132259 |
| PRICKLE2 | 1.06546493 | 1.001081882 | 1.133988675 | 0.04615584 |
| MAPK10 | 1.516187389 | 1.126541383 | 2.04060342 | 0.006029912 |
| CDO1 | 1.209294388 | 1.042199313 | 1.403179698 | 0.012253839 |
| PTCH2 | 1.061214825 | 1.015932462 | 1.108515523 | 0.007575781 |
| DAAM2 | 1.050911313 | 1.008504041 | 1.095101797 | 0.018132133 |
| ADAMTS8 | 1.07877286 | 1.027102683 | 1.133042395 | 0.00246318 |
| RNF180 | 1.402579675 | 1.092957549 | 1.799914137 | 0.00785051 |
| MAP6 | 1.138040505 | 1.015377077 | 1.275522386 | 0.026268795 |
| PPP1R1B | 1.000684565 | 1.000063578 | 1.001305937 | 0.030718693 |
| PTGFR | 1.181066323 | 1.007563249 | 1.384446743 | 0.040081071 |
| NAP1L3 | 1.362955155 | 1.087138895 | 1.708748315 | 0.007270383 |
| ACTA2 | 1.000810309 | 1.000093068 | 1.001528065 | 0.026802093 |
| C14orf132 | 1.052793707 | 1.003673667 | 1.104317694 | 0.034825405 |
