# Supplementary material for: Identification of a Gene Prognostic Model of Gastric Cancer Based on Analysis of Tumor Mutation Burden
Source: Pathol Oncol Res. 2021 Sep 10;27:1609852. doi: 10.3389/pore.2021.1609852 (PMC8460769; doi:10.3389/pore.2021.1609852)
Supplement: Supplementary file 3 [file table3.docx]

| Table S3 Correlation of 11 hub genes expression with immune infiltration level | | | | | | | | | | | | |
| --- | --- | --- | --- | --- | --- | --- | --- | --- | --- | --- | --- | --- |
| Gene | B Cell | | CD8+ T Cell | | CD4+ T Cell | | Macrophage | | Neutrophil | | Dendritic Cell | |
|  | Cor | p | Cor | p | Cor | p | Cor | p | Cor | p | Cor | p |
| SCGB3A1 | 0.143285317 | 0.005827659 | -0.009229069 | 0.859565115 | 0.067456837 | 0.197892956 | 0.009275763 | 0.858862012 | -0.063002041 | 0.226043759 | -0.060501629 | 0.245042008 |
| UPK1B | 0.215198226 | 3.06E-05 | 0.033988196 | 0.514561044 | 0.080131613 | 0.125960872 | 0.066695534 | 0.200545516 | 0.025798313 | 0.620376927 | 0.023179806 | 0.656299696 |
| XG | 0.040237317 | 0.440930233 | -0.001591636 | 0.975658599 | 0.227206977 | 1.14E-05 | 0.258985118 | 4.39E-07 | 0.040555873 | 0.436070593 | 0.060086902 | 0.248297959 |
| CCL21 | 0.09746878 | 0.061425879 | 0.364414605 | 4.61E-13 | 0.426706147 | 1.26E-17 | 0.459695259 | 9.59E-21 | 0.364032362 | 4.56E-13 | 0.467113916 | 1.66E-21 |
| CDC6 | -0.188014758 | 0.000281416 | -0.196834126 | 0.000138566 | -0.267534154 | 2.04E-07 | -0.386425964 | 1.26E-14 | -0.152313337 | 0.003271119 | -0.249948963 | 1.08E-06 |
| PLA2G5 | -0.040259857 | 0.440674114 | 0.199820336 | 0.000108964 | 0.264024629 | 2.97E-07 | 0.475664396 | 2.75E-22 | 0.232130915 | 6.24E-06 | 0.338288872 | 2.20E-11 |
| LAMP5 | -0.021033655 | 0.687156412 | 0.245853631 | 1.69E-06 | 0.33086701 | 8.47E-11 | 0.523651441 | 1.93E-27 | 0.27294651 | 9.23E-08 | 0.444561913 | 2.10E-19 |
| NLGN4Y | 0.083930891 | 0.107478467 | 0.076370357 | 0.142598026 | 0.170805565 | 0.001035625 | 0.240473236 | 2.89E-06 | 0.072019131 | 0.166265544 | 0.152990976 | 0.003133986 |
| NPR3 | 0.067553018 | 0.195414149 | 0.141973949 | 0.006227558 | 0.271415757 | 1.33E-07 | 0.499371651 | 9.89E-25 | 0.140025427 | 0.006906909 | 0.291747421 | 1.03E-08 |
| CPA3 | 0.150984138 | 0.00364734 | 0.368130738 | 2.56E-13 | 0.347826461 | 7.57E-12 | 0.508383631 | 1.03E-25 | 0.350692679 | 3.55E-12 | 0.450397956 | 6.21E-20 |
| PPP1R1B | 0.001115529 | 0.982961737 | -0.229227097 | 8.44E-06 | -0.151364508 | 0.003700673 | -0.207285311 | 5.88E-05 | -0.162124345 | 0.001730593 | -0.263929933 | 2.49E-07 |

Cor: Correlation coefficient.
